# A comparison of the low temperature transcriptomes of two tomato genotypes that differ in freezing tolerance: *Solanum lycopersicum* and *Solanum habrochaites*

**DOI:** 10.1186/s12870-015-0521-6

**Published:** 2015-06-06

**Authors:** Hongyu Chen, Xiuling Chen, Dong Chen, Jingfu Li, Yi Zhang, Aoxue Wang

**Affiliations:** Heilongjiang Provincial Key University Laboratory of Agricultural Functional Genes, College of Life Science, Northeast Agricultural University, Harbin, 150030 China; College of Horticulture, Northeast Agricultural University, Harbin, 150030 China; ABLife, Inc, Wuhan, 430075 China

**Keywords:** Cold, Transcriptome, RNA sequencing, Alternative splicing, microRNA

## Abstract

**Background:**

*Solanum lycopersicum* and *Solanum habrochaites* are closely related plant species; however, their cold tolerance capacities are different. The wild species *S. habrochaites* is more cold tolerant than the cultivated species *S. lycopersicum*.

**Results:**

The transcriptomes of *S. lycopersicum* and *S. habrochaites* leaf tissues under cold stress were studied using Illumina high-throughput RNA sequencing. The results showed that more than 200 million reads could be mapped to identify genes, microRNAs (miRNAs), and alternative splicing (AS) events to confirm the transcript abundance under cold stress. The results indicated that 21 % and 23 % of genes were differentially expressed in the cultivated and wild tomato species, respectively, and a series of changes in *S. lycopersicum* and *S. habrochaites* transcriptomes occur when plants are moved from warm to cold conditions. Moreover, the gene expression patterns for *S. lycopersicum* and *S. habrochaites* were dissimilar; however, there were some overlapping genes that were regulated by low temperature in both tomato species. An AS analysis identified 75,885 novel splice junctions among 172,910 total splice junctions, which suggested that the relative abundance of alternative intron isoforms in *S. lycopersicum* and *S. habrochaites* shifted significantly under cold stress. In addition, we identified 89 miRNA sequences that may regulate relevant target genes. Our data indicated that some miRNAs (e.g., miR159, miR319, and miR6022) play roles in the response to cold stress.

**Conclusions:**

Differences in gene expression, AS events, and miRNAs under cold stress may contribute to the observed differences in cold tolerance of these two tomato species.

**Electronic supplementary material:**

The online version of this article (doi:10.1186/s12870-015-0521-6) contains supplementary material, which is available to authorized users.

## Background

Some plants increase their cold tolerance to deal with low temperatures; this phenomenon is termed cold acclimation. During this process, various biochemical and physiological changes occur in plants, which make plants more cold tolerant. Plants have different abilities to deal with low temperatures. Plants that have adapted to cold environments increase their cold tolerance in response to low but nonfreezing temperatures. By contrast, plants that have adapted to subtropical and tropical climates, such as maize, rice, and tomato, generally have little cold tolerance and are unable to acclimate to cold temperatures [1].

In recent years, many cold-regulated genes have been identified in plants under cold stress. The C-repeat binding factor (CBF) cold-responsive pathway is considered the best-known cold tolerance pathway in plants [2, 3]. There are three CBF/dehydration-responsive element binding factor 1 (DREB1) family members, including CBF1, CBF2, and CBF3 (DREB1b, DREB1c, and DREB1a, respectively) [4–6], encoding DNA-binding proteins of the APETALA2 (AP2)/ethylene response factor family [7]. Overexpression of CBF1, CBF2, or CBF3 of *Arabidopsis thaliana* caused an increase in cold tolerance in the absence of cold stress in plants, showing that the CBF genes improve cold tolerance [8–11]. Studies have shown that overexpression of CBF genes increases the cold tolerance of *A. thaliana* [8,9], *Brassica napus* [12], poplar [13], and potato [14], but not in tomato [1].

The roles of cold-regulated genes in plant cold acclimation show that differential expression of genes is related to different cold adaption abilities of plants. In *A. thaliana* and *Chorispora bungeana*, many alterations in gene expression begin within minutes of transferring plants to a low temperature [15–19]. Moreover, some studies have demonstrated that differential expression of a cold-responsive gene is caused by differences in cold tolerance in plants [20, 21]. For example, there are considerable differences in cold-responsive genes in *Solanum tuberosum* and *Solanum commersonii*, which are closely related but have different cold tolerances [22].

A large number of cold-related genes have been identified using transcript analysis techniques, and the products of cold-related genes, including regulatory proteins and functional proteins, are thought to play key roles in gene expression and signal transduction [3, 15, 17, 23–28]. The expression and alternative splicing (AS) of some serine/arginine-rich (SR) genes, which encode splicing factor proteins that are vital for splicing and constitutive expression, vary under cold stress [29–33]. Cold stress affects the expression of splicing factors; therefore, the splicing of precursor-mRNAs (pre-mRNAs) of other genes are altered under cold stress. AS of pre-mRNA is an important mechanism for increasing transcriptome and proteome variety in eukaryotes. AS has been confirmed widely at the functional level in *A. thaliana*, rice, and maize [34–36]. AS may be regulated spatially and developmentally under environmental stress [33, 37–39]; thus, AS could play an important role under cold stress or other abiotic stress.

MicroRNAs (miRNAs) have been discovered in plants [40–42], changing our perception of the mechanisms of gene expression, transcription, and translation. In plants, 21–24 miRNAs are negative regulators of gene expression [43]. The pool of miRNAs in plants is highly diverse [44–46]. Many studies have indicated that the key role of miRNAs is regulating organ development and biological processes [47–49]. MiRNAs are also associated with abiotic stress responses [50–52]. In accordance with their regulatory roles, many miRNAs target genes that have roles in developmental patterning and show unique development-specific, tissue-specific, and stress-induced expression patterns [47, 53, 54]. However, to date, only 44 annotated tomato miRNAs have been deposited in the miRBase database v19.0, and only a few miRNA targets have been confirmed experimentally. At present, it is unknown whether important regulators such as miRNAs play a vital role in tomato’s response to cold stress.

The cultivated tomato species (*Solanum lycopersicum*) suffers from cold stress at all stages of growth and development; by contrast, the wild tomato species (*Solanum habrochaites*) grows well at low temperatures [55–57]. To understand the molecular basis underlying why *S. habrochaites* can acclimate to cold and survive freezing temperatures, whereas *S. lycopersicum* cannot, we report the results of an RNA sequencing (RNA-seq) transcriptome and miRNA analysis of RNA populations obtained from cold-treated leaves of the two plants. The results showed that many changes in the *S. lycopersicum* and *S. habrochaites* transcriptomes occur in plants transferred from warm to cold conditions. We predicted that at least 21 % and 23 % of genes were cold responsive in *S. lycopersicum* and *S. habrochaites*, respectively. A gene ontology (GO) term enrichment analysis of the data indicated that many GO terms (“abiotic stimulus response”, “ethylene stimulus response”) were significantly enriched in the cold-responsive genes between the two species. Our data also provided an evaluation of AS between *S. lycopersicum* and *S. habrochaites*. RNA-seq identified many annotated introns, known AS, and 75,885 novel splice junctions. We identified 89 miRNA sequences and 423 targets of 83 miRNAs. Our data showed that some miRNAs (e.g., miR159, miR319, and miR6022) play important roles under cold stress in the two species. These results provided a new insight into the roles of miRNAs under cold stress in these two closely related species under cold stress. Thus, the differences in gene expression, AS events, and miRNAs under cold stress may contribute to the differences in the cold tolerance between *S. lycopersicum* and *S. habrochaites*.

## Results

### Phenotypic and physiological responses to cold stress

*Solanum lycopersicum* and *S. habrochaites* leaf tissue were chosen to study cold responses. The degree of cold stress was identified by malondialdehyde (MDA) content, proline content, peroxidase (POD) activity, and catalase (CAT) activity exchange in the leaves. *S. habrochaites* exhibited less severe wilting than *S. lycopersicum* after 10 days of treatment at 4 °C (Additional file 20: Figure S1A–D). Cold stress caused significant increased MDA content, proline content, POD activity, and CAT activity in these plants (Additional file 20: Figure S1E–H).

### *Solanum lycopersicum* and *S. habrochaites* transcriptome analyses

The transcriptomes of *S. lycopersicum* (C) and *S. habrochaites* (Tsh) under cold stress were analyzed by RNA-seq using the Illumina Genome Analyzer II. After growth at 25 °C for 12 weeks, plants were moved to 4 °C for 0, 1, and 12 h, and the total RNA from leaves was extracted and analyzed. More than 200 million reads were produced, with approximately 33.3 million reads from each sample. We aligned the reads to the entire reference genome sequence of tomato (version SL2.40; http://solgenomics.net/) using the TopHat tool. The tolerance was set to allow two mismatches at most for reads in each alignment. Using these criteria, 96.32–97.21 % of the reads mapped uniquely to a genomic location, and 2.79–3.68 % of the reads were filtered out as multiple-mapped or low-quality reads. Alignment of the reads to tomato cDNAs demonstrated that 70 % of the tomato genome-annotated cDNAs had a sequence represented by Illumina RNA-seq reads (Table 1). Compared with the annotated tomato genome, the RNA-seq data revealed that 92.5–95 % of the reads that matched to the genome mapped to annotated genic regions, but only 5–7.5 % of the reads mapped to intergenic regions (Additional file 20: Figure S2). The depth of coverage along the length of the transcripts reduced towards the 5′ termini for RNA-seq data derived from the full-length cDNA libraries (Additional file 20: Figure S3). De novo assembly was performed using the Trinity method with default parameters [58]. Overviews of the assembly results are shown in Additional file 18 and Additional file 19. The reads were assembled into 68,051 non-redundant unigenes (>200 bp) in *S. habrochaites*.

**Table 1:** Number of reads sequenced and mapped with TopHat

Sample	Raw Data	Clean Reads	Unique Mapped	Multiple Mapped	Total Mapped	Expressed Genes (Mapped Reads Number > 0)	Expressed Genes (Mapped Reads Number > 10)
C0	35,588,324	30,268,069(85.05 %)	25,796,713(97.15 %)	756,667(2.85 %)	26,553,380(87.75 %)	24,776(71.35 %)	20,244(81.71 %)
C1	47,221,174	42,718,256(90.46 %)	32,505,159(96.96 %)	1,018,137(3.04 %)	33,523,296(78.49 %)	25,242(72.69 %)	20,515(81.27 %)
C12	34,010,526	29,504,403(86.75 %)	24,478,334(97.21 %)	702,267(2.79 %)	25,180,601(85.37 %)	24,438(70.37 %)	19,843(81.20 %)
Tsh0	36,781,738	32,613,936(88.67 %)	23,386,815(96.45 %)	860,942(3.55 %)	24,247,757(74.37 %)	25,231(72.66 %)	20,155(79.88 %)
Tsh1	35,295,488	31,895,199(90.37 %)	21,154,377(96.32 %)	807,256(3.68 %)	21,961,633(68.87 %)	25,074(72.20 %)	19,775(78.87 %)
Tsh12	37,022,418	33,210,743(89.70 %)	23,125,182(96.68 %)	793,404(3.32 %)	23,918,586(72.04 %)	24,845(71.54 %)	19,698(79.28 %)
C0 (Small RNA)	5,146,411	4,329,523(84.13 %)	1,591,965(38.20 %)	2,575,134(61.80 %)	4,167,099(96.25 %)		
C1 (Small RNA)	6,430,561	5,210,088(81.02 %)	2,287,038(45.28 %)	2,764,182(54.72 %)	5,051,220(96.95 %)		
C12 (Small RNA)	6,841,937	4,584,516(67.01 %)	1,438,066(32.84 %)	2,941,450(67.16 %)	4,379,516(95.53 %)		
Tsh0 (Small RNA)	4,375,096	3,355,947(76.71 %)	757,007 (26.23 %)	2,128,856(73.77 %)	2,885,863(85.99 %)		
Tsh1 (Small RNA)	4,459,693	3,635,142(81.51 %)	935,282(29.92 %)	2,190,299(70.08 %)	3,125,581(85.98 %)		
Tsh12 (Small RNA)	5,089,855	4,202,130(82.56 %)	1,011,253(27.90 %)	2,613,036(72.10 %)	3,624,289(86.25 %)		

The number of unique mapping reads plus multiple mapping reads equals the total number of alignments. C0, C1 and C12 represent *S. lycopersicum* cold treatment for 0 h, 1 h and 12 h, respectively; Tsh0, Tsh1 and Tsh12 indicate *S. habrochaites* cold treatment for 0 h, 1 h and 12 h, respectively

To verify the RNA-seq data, some genes whose expressions increased, some that decreased, and some that showed no change in abundance were chosen for real-time PCR (qPCR) under cold stress. The results of RNA-seq and qPCR were similar (Additional file 20: Figure S8), showing the same general expression trends by qPCR as were revealed by RNA-seq.

To identify *S. lycopersicum* and *S. habrochaites* miRNAs that affect gene regulation under cold stress, six miRNA libraries were constructed from the leaves of *S. lycopersicum* and *S. habrochaites* that were or were not treated with cold. The six miRNA libraries were sequenced using high-throughput RNA-seq and yielded approximately 5.4 million raw reads in each sample. We excluded the poor-quality reads and those whose length was smaller than 14 nucleotides from further analysis. Finally, we obtained approximate 4.2 million non-redundant reads (14–24 nucleotides) in each sample (Table 1).

### Differential expression and GO enrichment

**Fig. 1:**
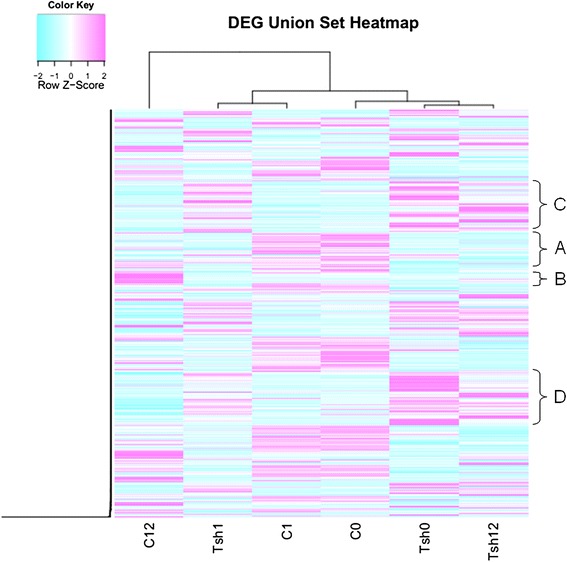
Hierarchical clustering of *S. lycopersicum* (C) and *S. habrochaites* (Tsh) transcripts at 0, 1, and 12 h of cold treatment at 4 °C

To study the impact of cold stress on gene expression in *S. lycopersicum* (C) and *S. habrochaites* (Tsh), the transcript abundance of each gene was identified by the reads per kilobase of transcript per million reads mapped method (Additional file 1, Additional file 20: Figure S4). To compare the transcriptomes in *S. lycopersicum* and *S. habrochaites* under cold stress, a heat map was generated to present the transcript abundance for all differentially expressed genes (DEGs) under cold stress at 0, 1, and 12 h (Fig. 1, Additional file 20: Figure S5–7). The results showed that a series of changes in gene expression in *S. lycopersicum* and *S. habrochaites* occur when plants are moved from warm to cold conditions. Moreover, the gene expression patterns for *S. lycopersicum* and *S. habrochaites* were dissimilar. For example, cluster A genes were little affected at 1 h in *S. lycopersicum* and returned to low transcript abundance levels at 12 h of cold stress; cluster B genes were unaffected in *S. lycopersicum* at 1 h of cold stress, but were highly increased at 12 h; cluster C or D genes were little affected after cold stress in *S. lycopersicum,* but were affected in *S. habrochaites* (Fig. 1).

**Fig. 2:**
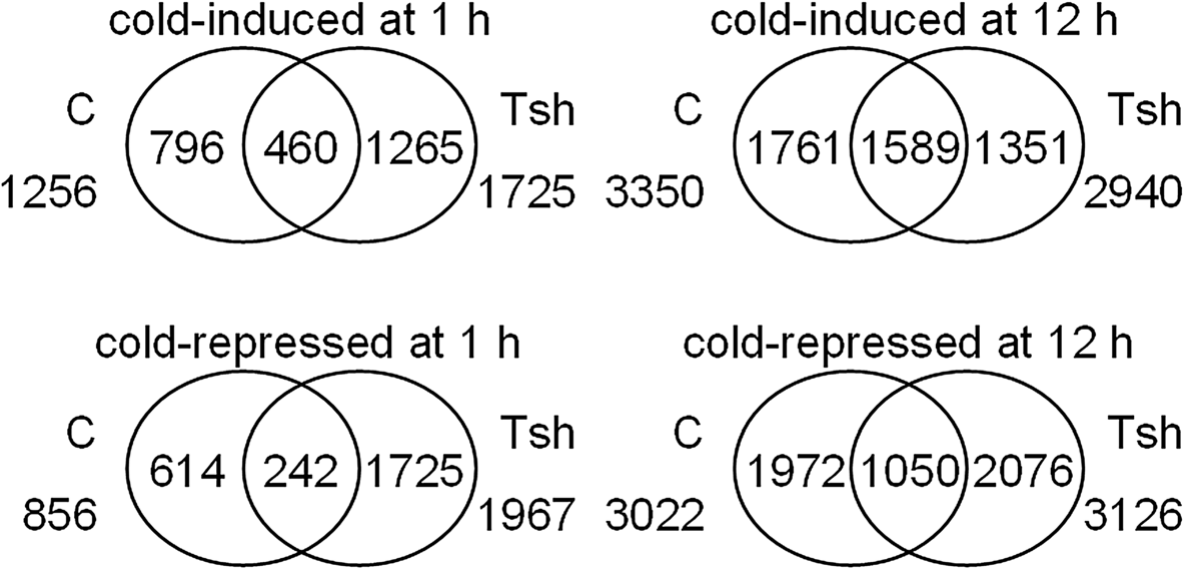
The number of total ESTs that were either cold-induced or cold-repressed by 2-fold change in *S. lycopersicum* (C) and *S. habrochaites* (Tsh). The results from 0, 1, and 12 h of cold treatment at 4 °C

We used a threshold of a minimum 2-fold change in abundance between any two time points to define DEGs in the following analysis (Fig. 2, Table 2, Additional file 3). The results showed that ~4 % (sample C1 *vs*. C0), ~10 % (sample C12 *vs*. C0), ~5 % (sample Tsh1 *vs*. Tsh0), and ~8 % (sample Tsh12 *vs*. Tsh0) of the unigenes were cold induced; and ~2 % (sample C1 *vs*. C0), ~9 % (sample C12 *vs*. C0), ~6 % (sample Tsh1 *vs*. Tsh0), and ~9 % (sample Tsh12 *vs*. Tsh0) were cold repressed. In *S. lycopersicum*, transcripts for 1,256 and 3,350 unigenes increased at 1 and 12 h, respectively, and 804 unigenes increased at both time points tested; transcripts for 856 and 3,022 unigenes decreased at 1 and 12 h, respectively, and 339 unigenes decreased at both time points tested (Fig. 2, Table 2, Additional file 3, Additional file 4). In *S. habrochaites*, transcripts for 1,725 and 2,940 unigenes increased at 1 and 12 h, respectively, and 722 unigenes increased at both time points tested; transcripts for 1,967 and 3,126 unigenes decreased at 1 and 12 h, respectively, and 1,000 unigenes decreased at both time points tested. Moreover, in *S. habrochaites*, transcripts for 3,608, 2,813, and 3,549 unigenes increased at 0, 1, and 12 h, respectively, compared with *S. lycopersicum* at same time points; and transcripts for 3,897, 3,592, and 3,815 unigenes decreased at 0, 1, and 12 h, respectively. In sum, the gene expression profiles in *S. lycopersicum* and *S. habrochaites* changed under cold stress to different degrees; however, there were some overlapping genes that were regulated by low temperature in both tomato species.

**Table 2:** Total number of differentially expressed genes (DEG)

Group	Up Regulated Gene Numbe	Down Regulated Gene Number	Total
Tsh0 vs C0	3608	3897	7505
Tsh1 vs C1	2813	3592	6405
Tsh12 vs C12	3549	3815	7364
C1 vs C0	1256	856	2112
C12 vs C0	3350	3022	6372
C12 vs C1	3047	2748	5795
Tsh1 vs Tsh0	1725	1967	3692
Tsh12 vs Tsh0	2940	3126	6066
Tsh12 vs Tsh1	2962	2321	5283

C0, C1 and C12 represent *S. lycopersicum* cold treatment for 0 h, 1 h and 12 h, respectively; Tsh0, Tsh1 and Tsh12 indicate *S. habrochaites* cold treatment for 0 h, 1 h and 12 h, respectively

We analyzed the genes that we determined to be responsive to cold at 1 h. The GO terms enriched in each species were comparable (Additional file 3) and were generally related to “response to abiotic stimulus”. From the heat map, it was also obvious that *S. lycopersicum* was less affected by cold than *S. habrochaites* at 1 h. The expressions of genes that were enriched in GO categories corresponding to “cell wall metabolism” were increased under cold stress in *S. lycopersicum*, but the opposite result was observed in *S. habrochaites*. We observed a similar contrast in the GO category “response to organic substance”. In the GO categories “response to chitin”, “response to carbohydrate stimulus”, and “DNA-binding WRKY”, there was a significant enrichment in *S. lycopersicum*, but *S. habrochaites* showed no enrichment. For the categories “chloroplast”, “transit peptide”, “pentatricopeptide repeat”, “phenylpropanoid metabolic process”, “flavonoid metabolic process”, and “amino acid derivative biosynthetic process”, no significant enrichment was observed in *S. lycopersicum*, but enrichment was observed in *S. habrochaites* (Additional file 3).

We then compared responses to cold at 12 h. The analysis of GO terms for cold-regulated genes suggested that the categories “response to organic substance”, “response to endogenous stimulus”, “response to hormone stimulus”, “response to abscisic acid stimulus”, “pentatricopeptide repeat”, “response to abiotic stimulus”, “response to ethylene stimulus”, “serine/threonine-protein kinase”, “phenylpropanoid metabolic process”, “amino acid derivative biosynthetic process”, “lignin biosynthetic process”, and “flavonoid metabolic process” were enriched in both *S. lycopersicum* and *S. habrochaites* (Additional file 3). In the case of the GO category “UDP-glucuronosyl/UDP-glucosyltransferase”, there was significant enrichment for *S. lycopersicum*, but not for *S. habrochaites.*

### Alternative splicing in *S. lycopersicum* and *S. habrochaites*

To study the effect of cold stress on AS in *S. lycopersicum* and *S. habrochaites*, we compared splicing events between the two tomato genotypes. We identified splice junctions using the TopHat software [59]. Collectively, using RNA-seq data, we identified 105,663, 109,251, 102,316, 106,690, 104,440, and 105,323 splice junctions in samples C0, C1, C12, Tsh0, Tsh1, and Tsh12 with 21,548, 25,492, 22,870, 20,909, 19,957, and 23,179 novel junctions, respectively (Additional file 5). We categorized each AS event using the primary known types of AS and the sequencing data (Table 3, Additional file 6). As previously reported [60–62], we found that intron retention was the primary type of AS.

**Table 3:** Classification of all detected alternative splicing events in tomato

Samples	3pMXE	5pMXE	A3SS	A3SS and ES	A5SS	A5SS and ES	Cassette Exon	ESx	IntronR	MXE	Total	Detected novel junctionx	AS/100 novel SJ
C0	187	206	1220	35	941	35	136	328	3079	24	6191	21548	28.73
C1	190	204	1116	33	919	30	104	376	3361	18	6351	25492	24.91
C12	187	208	1138	43	933	38	86	1071	3983	17	7704	22870	33.69
Tsh0	201	249	1055	24	846	46	99	317	1931	16	4784	20909	22.88
Tsh1	183	213	1007	28	798	35	60	326	1970	5	4625	19957	23.17
Tsh12	188	233	1096	50	858	47	57	1143	2951	10	6633	23179	28.62
Total	396	506	2953	102	2230	94	317	2091	8211	57	16957	75885	22.35

C0, C1 and C12 represent *S. lycopersicum* cold treatment for 0 h, 1 h and 12 h, respectively; Tsh0, Tsh1 and Tsh12 indicate *S. habrochaites* cold treatment for 0 h, 1 h and 12 h, respectively

**Fig. 3:**
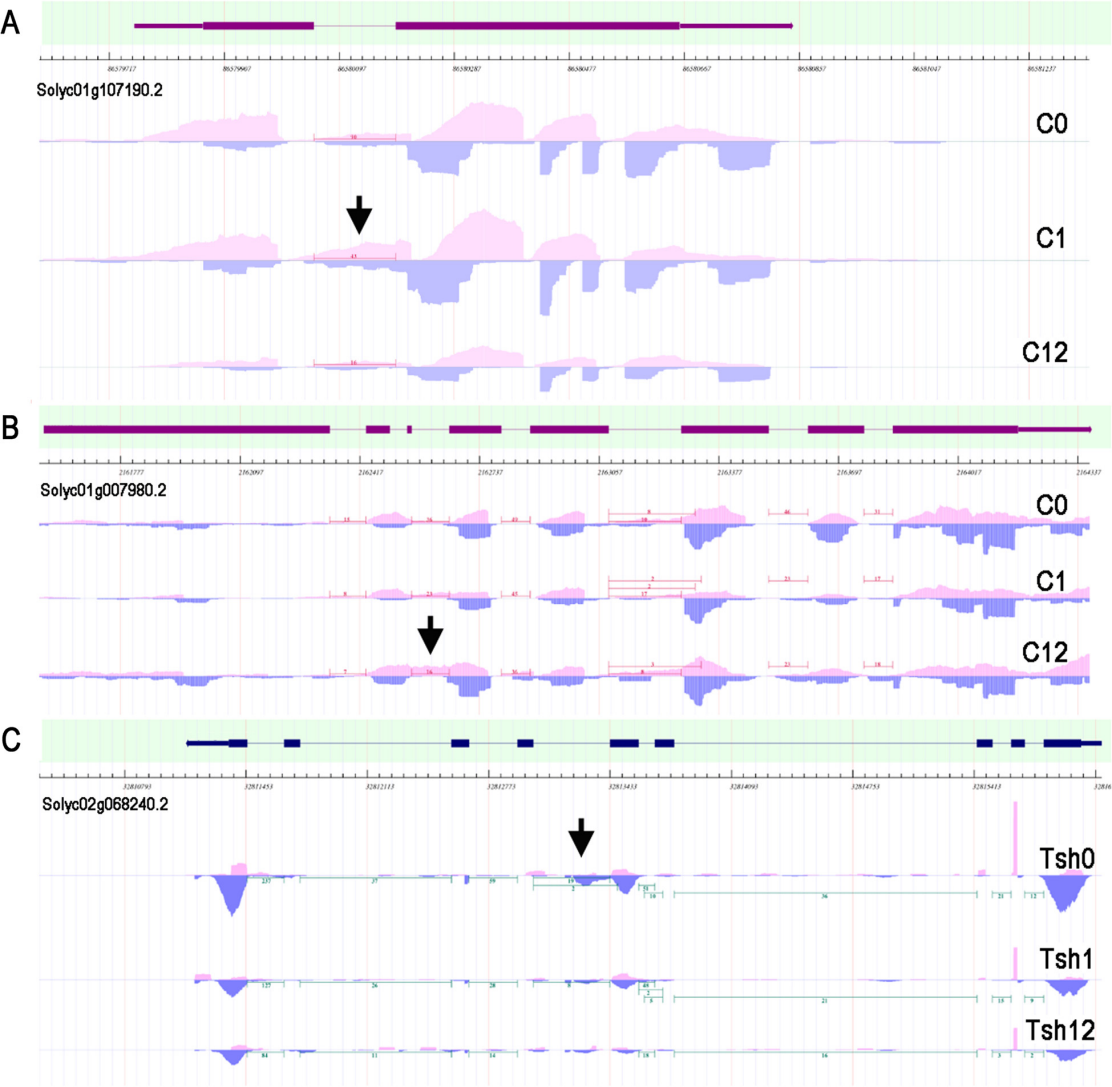
Identification of alternative splicing in the LOB domain-containing protein 37 (Solyc01g107190.2) (**a**), cysteine-rich RLK 2 (Solyc01g007980.2) (**b**), and diacylglycerol acyltransferase 2 (Solyc02g068240.2) (**c**) transcripts. Changes in read density coverage are indicated by pink (forward reads) and blue (reverse reads). The intron retention events are indicated by an arrow

Illuminative examples, including intron retention in the LOB domain protein 38 (Solyc01g107190.2) (Fig. 3a) and receptor-like protein (RLK) (Solyc01g007980.2) (Fig. 3b), are shown in Fig. 3. The TopHat-generated *S. lycopersicum* LOB domain protein 38 mRNA model predicted a distinct AS event yielding a splice isoform that retains full-length intron 1 (Fig. 3a, Additional file 7). An analysis of RLK, a putative resistance protein with an antifungal domain, provides another example of an IntronR event in plants. The depth of coverage of the intron 3 splice junction was confirmed by RNA-seq (Fig. 3b, Additional file 7). Dense micro-read coverage of intron 3 in the RLK transcript contrasted with the low coverage of other introns, indicating that intron 3 may be retained in some mature RLK transcripts (Fig. 3b).

We tried to identify differences in altered AS events between the two species at 0, 1, and 12 h of cold treatment at 4 °C. Then, 270 (sample Tsh0 *vs*. C0), 241 (sample Tsh1 *vs*. C1), 474 (sample Tsh12 *vs*. C12), 131 (sample C1 *vs*. C0), 575 (sample C12 *vs*. C0), 114 (sample Tsh1 *vs*. Tsh0), and 606 (sample Tsh12 *vs*. Tsh0) AS events were increased under cold stress; 204 (sample Tsh0 *vs*. C0), 237 (sample Tsh1 *v*s. C1), 412 (sample Tsh12 *vs*. C12), 119 (sample C1 *vs*. C0), 152 (sample C12 *vs*. C0), 122 (sample Tsh1 *vs*. Tsh0), and 130 (sample Tsh12 *vs*. Tsh0) events were decreased (Table 4). Table 4 shows that AS occurred more frequently in genes regulated in response to cold at 12 h than in genes at 1 h.

**Table 4:** Altered alternative splicing events between *S. lycopersicum* and *S. habrochaites*

Type	Total AS Number	Tsh0	Tsh1	Tsh12	C1	C12	C12	Tsh1	Tsh12	Tsh12
		Vs	Vs	Vs	Vs	Vs	Vs	Vs	Vs	Vs
		C0	C1	C12	C0	C0	C1	Tsh0	Tsh0	Tsh1
Increased under cold stress										
3pMXE	396	18	22	20	2	12	13	9	15	12
5pMXE	506	33	33	38	3	15	11	13	23	16
A3SS	2953	88	75	95	35	88	80	33	91	77
A3SSandES	102	2	1	3	0	2	1	1	6	7
A5SS	2230	53	35	64	18	66	59	23	70	54
A5SSandES	94	1	2	5	2	4	5	1	4	5
CassetteExon	317	16	11	8	12	14	11	4	12	13
ES	2091	27	35	206	34	300	291	19	358	324
MXE	57	5	2	3	5	5	4	1	3	3
IntronR	8211	27	25	32	20	69	86	10	24	32
Total	16957	270	241	474	131	575	561	114	606	543
Decreased under cold stress										
3pMXE	396	7	10	13	7	2	4	6	6	8
5pMXE	506	6	11	18	3	3	6	13	17	12
A3SS	2953	68	60	72	32	49	35	41	41	34
A3SSandES	102	2	0	1	0	1	0	0	1	1
A5SS	2230	39	54	54	15	42	27	14	22	24
A5SSandES	94	2	3	5	1	1	1	2	0	1
CassetteExon	317	19	21	15	18	18	19	18	20	11
ES	2091	31	48	182	19	17	17	17	14	13
MXE	57	10	5	5	5	8	6	2	1	1
IntronR	8211	20	25	47	19	11	22	9	8	6
Total	16957	204	237	412	119	152	137	122	130	111

C0, C1 and C12 represent *S. lycopersicum* cold treatment for 0 h, 1 h and 12 h, respectively; Tsh0, Tsh1 and Tsh12 indicate *S. habrochaites* cold treatment for 0 h, 1 h and 12 h, respectively

**Fig. 4:**
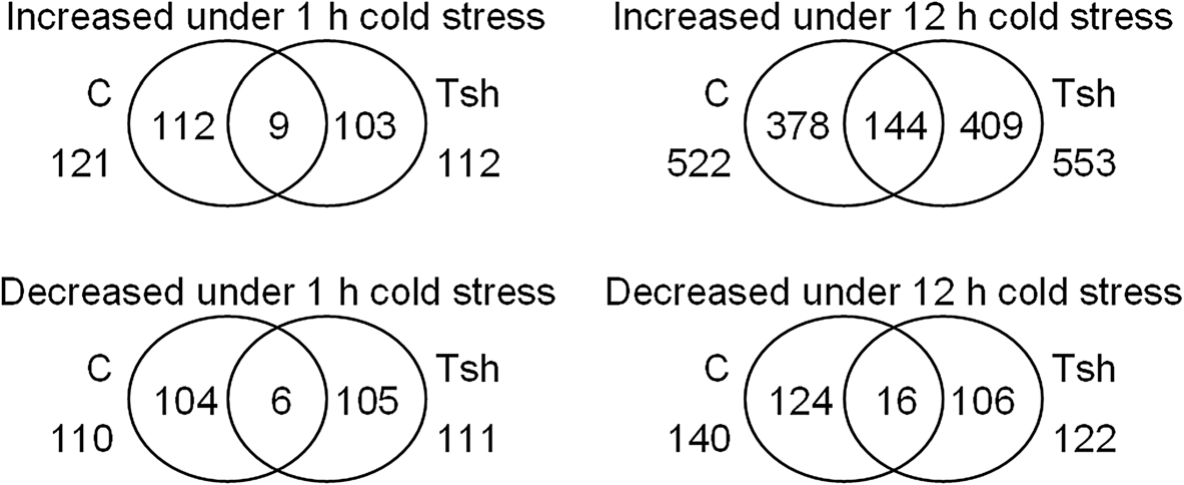
The total number of differentially alternative splicing genes (DASG) in *S. lycopersicum* (C) and *S. habrochaites* (Tsh) at 0, 1, and 12 h of cold treatment

Next, 121 (sample C1 *vs*. C0), 522 (sample C12 *vs*. C0), 112 (sample Tsh1 *vs*. Tsh0), and 553 (sample Tsh12 *vs*. Tsh0) of the AS genes were increased under cold stress; 110 (sample C1 *vs*. C0), 140 (sample C12 *vs*. C0), 111 (sample Tsh1 *vs*. Tsh0), and 122 (sample Tsh12 *vs*. Tsh0) of the genes were decreased (Fig. 4, Additional file 7). Certain AS events are associated with specific abiotic stress conditions [34]. An observation of individual events under cold stress showed that certain AS genes are cold associated (Additional file 8).

The AS event of diacylglycerol acyltransferase (Solyc02g068240.2) under cold stress is shown in Fig. 3c. The TopHat-generated *S. habrochaites* diacylglycerol acyltransferase mRNA model predicted a distinct AS event that yielded a splice isoform that retains intron 4 (Fig. 3c). Accumulation of the no IntronR 4-containing transcripts decreased approximately three-fold under cold treatment. Other examples of cold stress-associated AS genes (SR45a, SR30) are provided in Additional file 20: Figure S9.

We compared the functions of the AS genes that were regulated in response to cold at 1 h and 12 h with the DEGs. Cold-regulated differentially expressed AS genes overlapped with DEGs in *S. lycopersicum* (C) and *S. habrochaites* (Tsh), and these genes were in the GO categories “dephosphorylation” and “phosphoprotein phosphatase activity” (Additional file 8), suggesting these activities were present in both plants. In the case of the GO categories “detection of light stimulus”, “phenylpropanoid metabolic process”, “response to cadmium ion”, “phosphoinositide binding”, and “heat shock protein binding”, there was significant enrichment for *S. lycopersicum*, but *S. habrochaites* showed no enrichment. For the categories “carboxylic acid catabolic process”, “proteolysis”, “cell death”, “reproductive developmental process”, and “ethylene mediated signaling pathway”, no enrichment was observed in *S. lycopersicum*, but significant enrichment was observed in *S. habrochaites* (Additional file 8).

### Identification of single nucleotide polymorphisms (SNPs)

In comparison to the tomato reference genome, we identified 5,344 SNPs in *S. lycopersicum* ‘glamor’, and 3,625 of these SNPs were specific SNPs; and 154,870 SNPs were identified in *S. habrochaites* ‘LA1777’, and 153,157 of these SNPs were specific SNPs (Table 5, Additional file 9). We identified 696 (sample C1 *vs*. C0), 2,330 (sample C12 *vs*. C0), 1,157 (sample Tsh1 *vs*. Tsh0), and 2,183 (sample Tsh12 *vs*. Tsh0) genes that contained SNPs and were also cold induced; 463 (sample C1 *vs*. C0), 2,060 (sample C12 *vs*. C0), 1,452 (sample Tsh1 *vs*. Tsh0), and 2,311 (sample Tsh12 *vs*. Tsh0) genes that contained SNPs and were cold repressed (Additional file 10, Additional file 11). Genes that contained SNPs that were enriched in GO categories corresponding to “response to chitin” were increasingly expressed under cold stress at 1 h in *S. lycopersicum*, but not in *S. habrochaites*. Other examples of a similar contrast in GO categories are provided in Additional file 10.

**Table 5:** Statistical analysis of SNPs

Sample	SNP NO.	gene NO.	Specific SNP NO.	Specific gene NO.	Total SNP NO.	Total gene NO.
C	5344	2509	3625	1857	160214	19273
Tsh	154870	19090	153157	18905		

C stand for *S. lycopersicum*; Tsh stand for *S. habrochaites*

### Impact of cold stress on miRNAs in tomato

To identify miRNAs in tomato, we analyzed miRNAs by BLAST searches against the tomato genome sequence by BOWTIE (Additional file 20: Figure. S10). The novel miRNAs were then identified by the miRDeep2 tool. The sequences corresponding to the known non-coding RNAs (tRNAs, rRNAs, small nucleolar and small nuclear RNAs) were filtered out using BLASTn to search the Rfam database (http://rfam.xfam.org/) (Additional file 20: Figure S11). The remaining sequences were assigned as either other endogenous small RNAs or miRNA candidates and used for a fold-back structure prediction. We compared the unique miRNAs with the miRBase database (version 19.0). In this analysis, the miRNAs were required to show a perfect or nearly perfect match (mismatch ≤ 1) to known miRNAs. After these analyses, 112 unique miRNA were obtained as novel miRNA candidates (Additional file 12).

A large number of miRNA sequences were produced by Illumina sequencing, permitting us to confirm the relative abundance of miRNAs in tomato. To study the expression dynamics of miRNAs and their potential roles in gene expression regulation in *S. lycopersicum* and *S. habrochaites*, the transcript abundance of each miRNA was evaluated by transcripts per million (TPM). The TPM of the miRNAs varied from 0 to 27,670 (miR396a, sample C1), suggesting that the expression of miRNAs varied greatly in tomato (Additional file 13). MiR159 and miR396a were the most abundant miRNAs in the six sequencing datasets. According to the TPM, some miRNAs (miR159, miR396a, miR396b, miR482b, and miR6022) were highly expressed in tomato, with a TPM of greater than 100 each. MiR6027, miR171a, miR482, miR319, and miR1919a were moderately expressed and had a TPM between 10 and 100. MiR5303, miR169b, miR1916, miR171c, and miR395a represent miRNAs with low expression and a TPM of less than 10 (Additional file 13). Sequence analysis showed that the relative abundance of some members in one miRNA family changed considerably in tomato. For example, the TPM for miR396a was 9,433, whereas the TPM for miR396b was only 4,347 (Additional file 13).

**Fig. 5:**
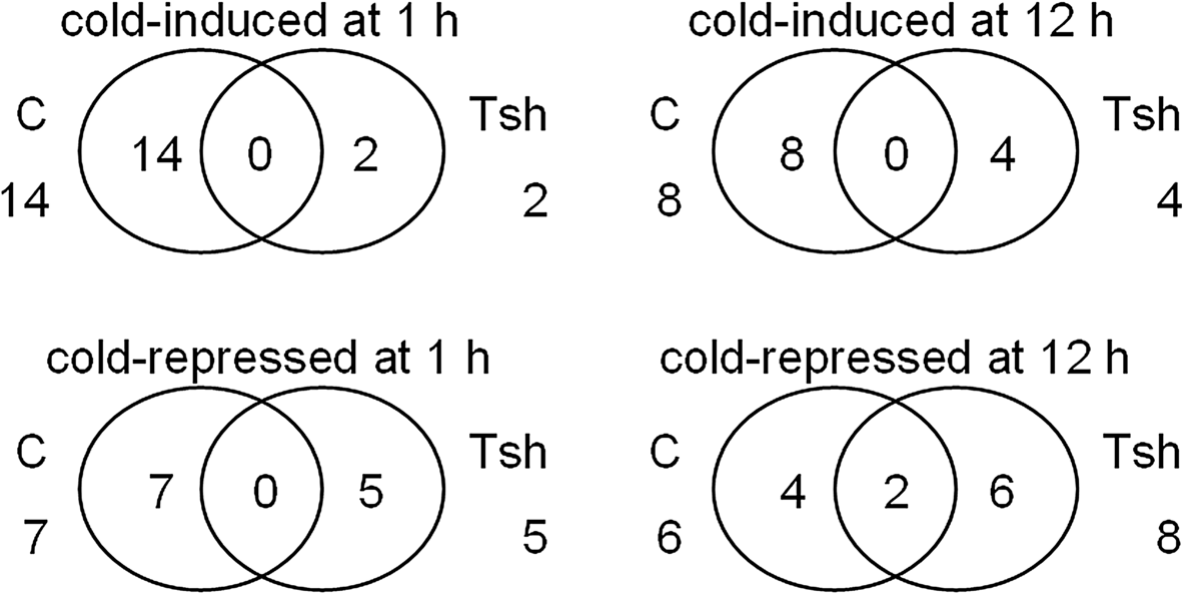
The total number of microRNAs (miRNAs) that were either cold-induced or cold-repressed in *S. lycopersicum* (C) and *S. habrochaites* (Tsh)

To detect the effect of cold stress on miRNAs, the expression of miRNAs in *S. lycopersicum* and *S. habrochaites* seedlings with and without cold treatment was examined. Fourteen (sample C1 *vs*. C0), eight (sample C12 *vs*. C0), two (sample Tsh1 *vs*. Tsh0), and four (sample Tsh12 *vs*. Tsh0) of the miRNAs were cold induced; seven (sample C1 *vs*. C0), six (sample C12 *vs*. C0), five (sample Tsh1 *vs*. Tsh0), and eight (sample Tsh12 *vs*. Tsh0) of the miRNAs were cold repressed (Fig. 5, Additional file 14). In response to cold treatment, the most significant change was observed for miR169c, whose expression level increased approximately 35-fold in sample C1 compared with C0. The expressions of some miRNAs in *S. habrochaites* were opposite to those in *S. lycopersicum* under cold stress. For example, miR1919a–miR1919c, and miR396b were upregulated under cold stress for 1 h in *S. lycopersicum*, whereas they were downregulated in *S. habrochaites* (Additional file 14). In contrast, miR172a and miR172b were downregulated by cold stress for 1 h in *S. lycopersicum*, while they upregulated in *S. habrochaites* by cold stress for 12 h.

**Fig. 6:**
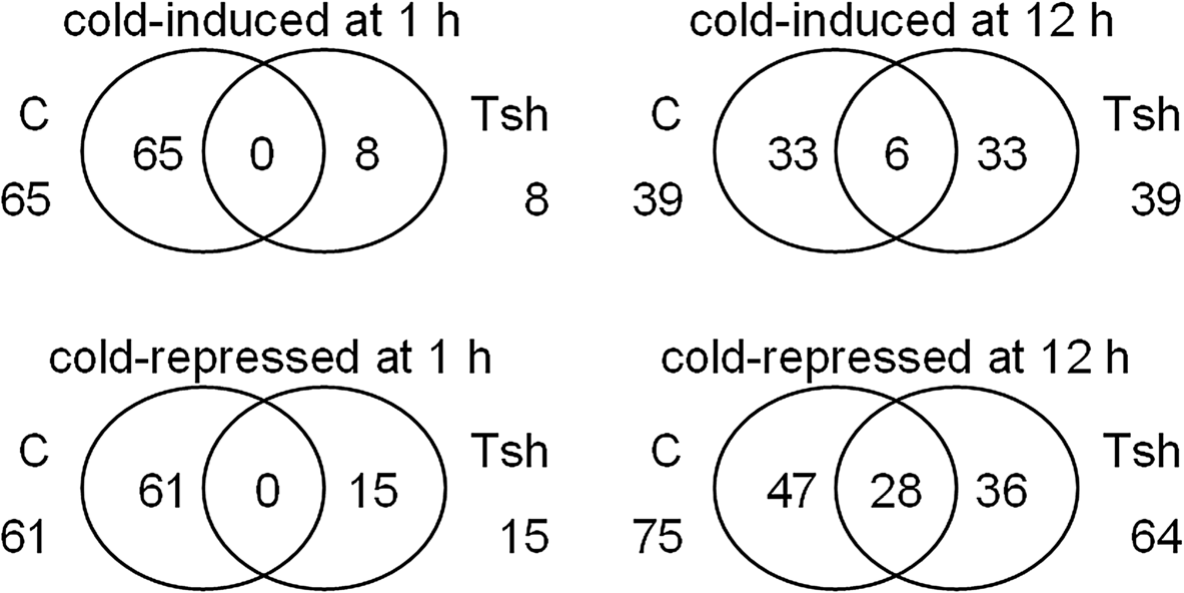
The total number of targets of differentially expressed microRNAs (DEmiRNAs) that were either cold-induced or cold-repressed in *S. lycopersicum* (C) and *S. habrochaites* (Tsh)

We used psRNATarget (http://plantgrn.noble.org/psRNATarget/) to predict targets for the miRNAs. For the miRNAs that were annotated as described above, we identified 423 mRNA targets (Fig. 6, Additional file 15). From Fig. 6 it was also evident that *S. lycopersicum* was more affected by 1 h of cold than *S. habrochaites*. To further characterize the role of the miRNAs in response to cold, we examined the target list for genes that could be related to the cold response and that were either induced or repressed by cold, based on our Illumina results (Additional file 17). For example, one of the predicted targets was the transcript of the homeodomain leucine zipper class I (HD-Zip I) protein (ATHB13, AT1G69780, Solyc02g087840.2) (Additional file 17). ATHB13 is induced in *S. lycopersicum* after cold treatment for 12 h based on our sequencing data (Additional file 3). The miRNA predicted to target ATHB13 is miR6022. Our sequencing data showed that miR6022 was downregulated in *S. lycopersicum* after cold stress for 12 h (Additional file 14). Based on our sequencing data, we did not find differential expression of miR6022 after cold treatment for 1 h in *S. lycopersicum*. The induction of ATHB13 under cold stress for 12 h correlates with miR6022 repression by cold, suggesting that ATHB13 levels are post-transcriptionally regulated by this miRNA in response to cold. Thus, miR6022/ATHB13 represents an abiotic stimulus module that could be important for the cold response in *S. lycopersicum* leaves. Other examples of cold stress-associated miRNAs (miR159, miR319) are provided in Additional file 17.

For comprehensive annotation, all putative target genes in each sample were analyzed by GO terms using the DAVID program. An analysis of GO enrichment for the targets revealed that target functions were enriched in many different biological processes (Additional file 16). Among the mRNA targets that were upregulated in response to cold at 1 h and 12 h, comparable cold-regulated mRNA target expression was observed between *S. lycopersicum* (C) and *S. habrochaites* (Tsh) in relation to the GO terms (Additional file 16), which included “ATP binding” and “nucleotide binding” in both species. In the case of the GO categories “leaf development”, “shoot development”, “CCAAT-binding factor”, “CBF”, “regulation of RNA metabolic process”, “cell death”, “gene silencing”, “immune response”, “flower development”, “ATPase activity”, and “leucine-rich repeat”, there was significant enrichment in *S. lycopersicum*, but not in *S. habrochaites*. For the categories “response to cold” and “hormone stimulus”, no enrichment was observed in *S. lycopersicum*, but significant enrichment was observed in *S. habrochaites* (Additional file 16).

Among the targets that were determined as downregulated in response to cold at 1 h and 12 h, an analysis of GO enrichment showed “defense response”, “ATP binding”, and “nucleotide binding” in both *S. lycopersicum* and *S. habrochaites*. In the case of the GO categories “leucine-rich repeat”, “reproductive structure development”, “meristem development”, “intracellular signaling cascade”, and “response to hormone stimulus”, there was no significant enrichment in *S. habrochaites*, but *S. lycopersicum* showed enrichment.

## Discussion

Plants have different abilities to deal with low temperatures. The cultivated tomato (*S. lycopersicum*) suffers from cold stress, but the wild species (*S. habrochaites*) does not [55–57]. RNA-seq of cold-stressed *S. lycopersicum* leaves and a comparison with the transcriptome of *S. habrochaites* are presented here. The results revealed the effects of cold stress on transcript abundance in *S. lycopersicum* and *S. habrochaites*; 21 % and 23 % of transcripts in *S. lycopersicum* and *S. habrochaites*, respectively, are cold regulated. There is a large overlap in the genes that were cold responsive in both plant species, but the results indicated many differences in the cold-responsive genes of the two species (Figs. 1, 2). The diversity of GO categories that were enriched in cold-stressed *S. lycopersicum* and *S. habrochaites* (Additional file 3) indicated the complexity of the response.

For cold-regulated DEGs of *S. lycopersicum* and *S. habrochaites*, some similar clusters of GO categories “response to abiotic stimulus” was found in both plants (Additional file 3), confirming earlier observations [20]. However, in response to cold stress in *S. lycopersicum* at 1 h, many genes encoding proteins associated with the abiotic stimulus response showed increased transcript abundance, and a few genes showed decreased transcript abundance (Additional file 3).

The data also suggested that some GO terms overlap in cold-treated *S. habrochaites,* but not in *S. lycopersicum* (Additional file 3). Some photosynthesis-related GO terms were significantly enriched among the upregulated genes in *S. habrochaites* under cold stress at 1 h. The results indicated that photosynthesis-related genes in cold regulatory programs might contribute to transient cold tolerance of *S. habrochaites*. In 2012, Liu *et al*. studied the transcriptomes of *S. lycopersicum* and *S. habrochaites* under cold stress at 3 days using GeneChip [20]. Their experiments indicated that *S. lycopersicum* showed more severe inhibition of photosynthesis than *S. habrochaites* during chilling stress.

The data showed that the GO category “cell wall metabolism” was enriched with genes with increased expression in cold-treated *S. lycopersicum* at 1 h, but the opposite result was observed for cold-treated *S. habrochaites* at both 1 and 12 h (Additional file 3). A GO term enrichment analysis indicated that “cell wall metabolism” was significantly depressed in the long term by cold stress in *S. habrochaites*, but was transiently induced by cold stress in *S. lycopersicum*. The present findings in *S. habrochaites* are similar to those of Fowler and Thomashow [15], who used a microarray chip to analyze the transcriptome of Arabidopsis under cold stress.

Hormones are signaling molecules that play key roles in regulating gene expression under cold stress [15, 20]. RNA-seq analysis showed that many genes related to abscisic acid, ethylene, auxin, jasmonic acid, and gibberellin were regulated by cold stress in *S. lycopersicum* and *S. habrochaites* (Additional file 3). Two hormone-related GO terms, “response to abscisic acid stimulus” and “response to ethylene stimulus”, were significantly enriched among the differentially expressed genes in *S. lycopersicum* and *S. habrochaites* under cold stress (Additional file 3). In *S. lycopersicum*, our data show that ABA-related GO terms were significantly enriched among the upregulated genes under cold stress at 12 h and were not enriched at 1 h. In contrast, ABA-related GO terms were significantly enriched among the upregulated and downregulated genes under cold stress at multiple time points in *S. habrochaites*. The production of ethylene also has been associated with cold stress [63–65]. In *S. lycopersicum*, our data show that ethylene-related GO terms were significantly enriched among the upregulated genes under cold stress at multiple time points. In contrast, ethylene-related GO terms were only significantly enriched among the upregulated genes under cold stress at 12 h, but not at 1 h, in *S. habrochaites*.

**Fig. 7:**
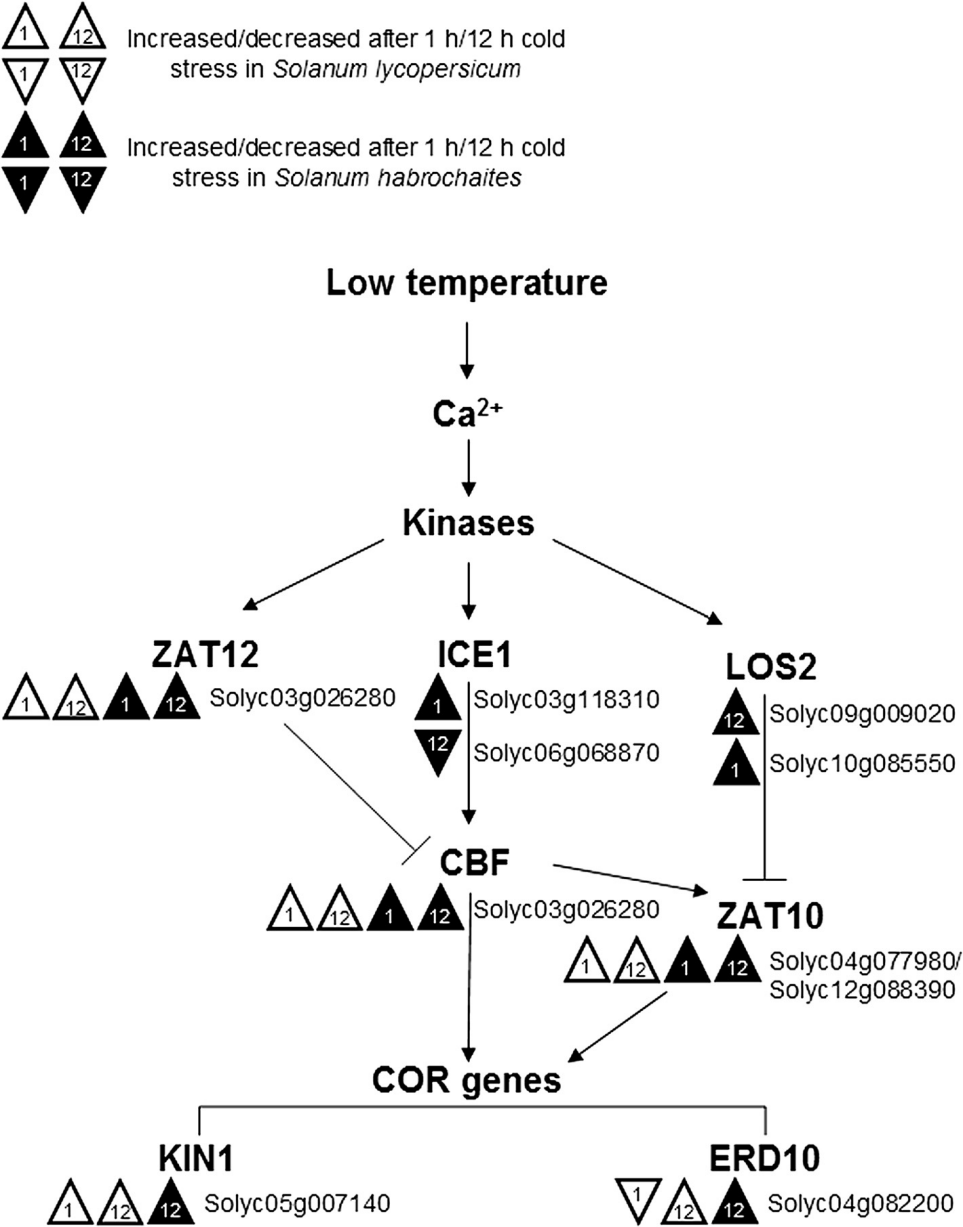
Diagram of cold-responsive transcriptional network in plant. Solid arrows indicate activation, whereas lines ending with a bar show negative regulation. Abbreviations: CBF, C-repeat binding factor (an AP2-type transcription factor); ICE1, inducer of CBF expression 1 (a MYC-type bHLH transcription factor); LOS2, low expression of osmotically responsive genes 2 (a bifunctional enolase with transcriptional repression activity); ZAT12, a zinc finger protein involved in high light and cold acclimation; ZAT10, related to Cys2/His2-type zinc-finger proteins found in higher plants; KIN1, encodes protein kinase APK2a; ERD10, encodes a gene induced by low temperature and dehydration

Transcription factors (TFs) play a key role in the regulation of gene expression under abiotic and biotic stresses in plants. The RNA-seq results showed that many TFs were regulated under cold stress in *S. lycopersicum* and *S. habrochaites*, and some members of the CBF were upregulated under cold stress at 1 h or 12 h (Fig. 7; Additional file 3). Two additional cold-regulated genes were found that encode transcription factors: a homolog of Cys2/His2-type zinc-finger protein (ZAT10) and a zinc finger protein involved in high light and cold stress (ZAT12). Indeed, the RNA-seq results showed that the transcript levels of a MYC-type bHLH transcription factor (ICE1) increased under cold stress at 1 h, and decrease after 12 h.

AS of pre-mRNA is an important mechanism to increase transcriptome and proteome variation in eukaryotes. Previous examinations of AS events under abiotic stress showed that some AS events are stress related [34, 66, 67]. Different coverage of mRNA isoforms in RNA-seq was observed under abiotic stress, likely reflecting the regulation of AS events. Here, we used RNA-seq to identify the AS events in *S. lycopersicum* and *S. habrochaites* that differed under normal conditions and cold stress treatment. Compared with other methods, RNA-seq supplies a wide and deep sequencing of the transcriptome, providing experimental confirmation of splice junctions and AS events with low false-positive rates. Our data provide an exceptional and impartial evaluation of AS in *S. lycopersicum* and *S. habrochaites*. The results were similar to those of Filichkin *et al.* [34], who used *A. thaliana* RNA-seq data to compare the specific abiotic stress transcriptomes of *A. thaliana*. The authors identified 6,000 novel AS events within the introns of 3,120 genes.

Our RNA-seq analysis of the *S. lycopersicum* and *S. habrochaites* transcriptomes suggests numerous genes with AS may be associated with cold stress (Additional file 7). The expressions of 121 (sample C1 *vs*. C0), 522 (sample C12 *vs*. C0), 112 (sample Tsh1 *vs*. Tsh0), and 553 (sample Tsh12 *vs*. Tsh0) AS genes were increased under cold stress; and the expressions of 110 (sample C1 *vs*. C0), 140 (sample C12 *vs*. C0), 111 (sample Tsh1 *vs*. Tsh0), and 122 (sample Tsh12 vs. Tsh0) genes decreased (Fig. 4, Additional file 7). Most of the genes identified as undergoing cold-induced AS were transcripts whose levels remained constant under cold stress. Thus, despite the lack of change in transcript expression level, their coding ability could be very different.

Recently, miRNAs have been identified as new players in plant tolerance to abiotic stress, such as cold, heat, high salinity, drought, oxidative, hypoxia, and UV B [68]. Many studies have attempted to understand the roles of miRNAs in the response to cold in several plants, including *A. thaliana* [69], *Brachypodium distachyon* [70], *Oryza sativa* [71], and *Populus trichocarpa* [72]. In this study, sequencing was used to confirm the genome-wide miRNA expression patterns of *S. lycopersicum* and *S. habrochaites* under cold stress.

Some miRNAs in different plant species present different expression patterns under cold stress. For example, the expression of miR172 was inhibited after cold stress at 1 h in *S. lycopersicum*, but was induced in *S. habrochaites* after cold stress at 12 h (Additional file 14). Additionally, miR172 was upregulated in *A. thaliana* [69] and *B. distachyon* [70]. Similarly, miR170/171 expression was upregulated under cold stress at 1 h in *S. lycopersicum*, while the transcript level of miR170/171 was decreased under cold stress at 12 h in *S. habrochaites*. The transcript level of miR170/171 was upregulated in *A. thaliana* [69] and downregulated in *Oryza sativa* [71] and *Populus trichocarpa* [72] in response to cold.

## Conclusions

*S. lycopersicum* and *S. habrochaites* are closely related plant species, but their cold tolerances are different. In recent years, our research group has investigated the cold-tolerance mechanisms of these plants at the physiological and molecular levels. Here, we studied the transcriptomes of cold stressed leaves of *S. lycopersicum* and *S. habrochaites*. We obtained 68,051 assembled unigenes, and many cold-regulated genes were detected, representing useful resources for gene cloning to improve cold tolerance of crops. Furthermore, the comparison of the functional networks of cold-regulated genes in *S. lycopersicum* and *S. habrochaites* provided information that could help us to identify the differences in cold-tolerance mechanisms between *S. lycopersicum* and *S. habrochaites*. We found that 21 % and 23 % of genes were differentially expressed between the cultivated and wild tomato species, respectively, when plants were transferred from warm to cold temperatures. An AS analysis suggested that the relative abundance of isoforms of *S. lycopersicum* and *S. habrochaites* significantly shifted under cold stress. In addition, certain miRNAs (e.g., miR159, miR319, and miR6022) play roles in the response to cold stress. Thus, differences in cold regulatory mechanisms may contribute to the differences in cold tolerance of these two tomato species.

## Methods

### Plant material and cold stress conditions

*S. habrochaites* LA1777 was supplied by Tomato Genetics Research Center (University of California, Davis, USA). *S. lycopersicum* ‘glamor’ and *S. habrochaites* were grow at 25 °C with 16-h light and 8-h dark cycles for 8 weeks before harvesting. To avoid changes caused by the circadian rhythm, all cold stress treatments were started at 4 °C at 12 PM under light and continued for 0 (untreated control), 1, and 12 h.

### Physiological responses to cold stress

The MDA content was assayed as described by Campos [73]. The free proline content was determined according to the method described by Zhang *et al.,* [70]. POD activities were determined as described by Quiroga [74]. CAT activity was assayed as described by Yao [75].

### Total RNA extraction and library preparation

The total RNA from leaves was extracted using the TRIzol reagent (Invitrogen) and digested with RQ1 DNase (Promega) to remove genomic DNA. The quality and integrity of the total RNA were detected using a SmartSpec plus Spectrophotometer (Bio-Rad) and 1.5 % agarose gel electrophoresis. Polyadenylated mRNAs were purified and concentrated using oligo(dT)-conjugated magnetic beads before being used for directional RNA-seq library preparation. Purified mRNAs were fragmented at 95 °C and subjected to end repair and 5′ adaptor ligation, followed by reverse transcription using randomized hexamers and an RT primer with a 3′ adaptor. Purified cDNAs were amplified, and 200–500 bp PCR products were quantified and purified. RNA-seq libraries were prepared and applied to an Illumina Genome Analyzer IIx system for 80 nt single-end sequencing by ABlife Inc. (Wuhan, China).

For small RNAs, 3 μg of total RNA was used for small RNA library preparation using the Balancer NGS Library Preparation Kit for small/microRNA (GnomeGen), following the manufacturer’s instructions. The purified small RNA libraries were quantified using a Qubit Fluorometer (Invitrogen) and used for cluster generation and 36 nt single-end sequencing analysis using the Illumina Genome Analyzer IIx system.

### Processing, mapping of Illumina reads, detection of alternative splicing, and de novo assembly

The adapter sequences and low-quality bases at the 3′ ends were removed from the RNA-seq reads generated by the Illumina Genome Analyzer. Reads whose lengths were more than 20 bp were used for further analysis. The reads were mapped to the tomato genome using TopHat [59], which allows the confirmation of AS events. In total, 172,910 junction sites were identified. We categorized the AS events into different types according to the exon structures, using the ABLas-1 package (ABlife Inc., Wuhan, China). These categories included exon skipping, intron retention, alternative 5′ splice site, alternative 3′ splice site, and mutually exclusive exons, as described by Wang et al. [76]. Reads were assembled separately from each *S. lycopersicoides* library using the Trinity method [58]. There are three software modules in Trinity: Inchworm, Chrysalis, and Butterfly, which were used to process the RNA-seq reads sequentially. First, the Inchworm program assembled the reads to contigs. Second, the Chrysalis program clustered the minimal overlapping contigs. Third, the Butterfly program constructed the transcripts. Finally, the multiple sequence alignment tool BLAST was used to cluster the transcripts by similarity of the right match length [49]. The coding sequences (CDS) of the unigenes were predicted using EMBOSS (http://emboss.sourceforge.net/) and the longest CDS was considered as the complete CDS of a unigene.

### Differential expression and GO enrichment

The expression level of genes were evaluated and normalized using the reads per kilobase of transcript per million reads mapped method [77]. Unigene expression was analyzed using the Bioconductor package with the edgeR and Bayseq methods. A very stringent cutoff, normalized fold change ≥2 or ≤0.5 and a P-value (P ≤0.01), was used to identify cold-regulated DEGs. A GO analysis was employed to predict gene function and calculate the functional distribution frequency, using the DAVID package (ABlife Inc., Wuhan, China).

### qPCR analysis

To validate the transcript abundance of genes measured by RNA-seq, we performed qPCR using Power SYBR Green Mastermix in an Applied Biosystems 7500 Real-Time PCR System. The RNAs from *S. habrochaites* and *S. lycopersicum* used in RNA-seq were reverse transcribed into cDNAs. The 14 primer pairs used are listed in Additional file 2. The ACTIN gene was used as a reference in these experiments. Three technical replicates were used for qPCR. The single amplicons were confirmed by melting curve analysis and gel electrophoresis of the final product. The cycle threshold (CT) value of each gene was normalized to the reference gene to detect the relative fold changes in each sample, which was calculated using the ΔΔCT method, as described previously [78].

### Identification of miRNAs in tomato

High-quality small RNA reads ranging from 14 to 24 nucleotides were acquired from the raw data. Adaptor sequences and low-quality tags were removed to detect known and novel miRNAs in tomato. Small RNA reads were used to search the Rfam database and NCBI database to remove non-coding RNAs, such as rRNA, tRNA, snRNA, and snoRNA. The remaining sequences were searched in the miRBase database v19.0, with no mismatch permitted, to identify conserved mature miRNA orthologs. Small RNAs that did not map to any miRNAs in miRBase database were analyzed as novel miRNAs using miRDeep2 (developed by ABlife Inc.).

### Availability of supporting data

The raw RNA-seq data supporting the result of this article is available in the Sequence Read Archive (SRA), with accession numbers SRX1013429 and SRX1014317.

## Additional files

Additional file 1:
**List of genes used to identify differentially expressed genes (DEG).**
Additional file 2:
**Specify primers used in expression analysis of selected genes.**
Additional file 3:
**GO term analysis of differentially expressed genes (DEG).**
Additional file 4:
**GO term analysis of co-differentially expressed genes (co-DEG).**
Additional file 5:
**All alternative splicing events in tomato.**
Additional file 6:
**Distribution of each class of alternative splicing events.**
Additional file 7:
**GO term analysis of differentially expressed alternative splicing genes (DASG).**
Additional file 8:
**Differentially expressed alternative splicing genes (DASG) overlap with differentially expressed genes (DEG).**
Additional file 9:
**GO term analysis of genes with SNPs.**
Additional file 10:
**SNP overlap with differentially expressed genes (DEG).**
Additional file 11:
**SNP overlap with co-differentially expressed genes (co-DEG).**
Additional file 12:
**List of novel miRNAs.**
Additional file 13:
**List of miRNAs to identify differentially expressed miRNAs.**
Additional file 14:
**Differentially expressed miRNAs.**
Additional file 15:
**List of differentially expressed miRNA targets.**
Additional file 16:
**GO term analysis of differentially expressed miRNA targets.**
Additional file 17:
**Differentially expressed miRNA targets overlap with differentially expressed genes (DEG).**
Additional file 18:
**Complete list of unigenes in** ***S. habrochaites*** **with BLASTX hits.**
Additional file 19:
**Unigene sequences were assembled from** ***S. habrochaites*** **reads.**
Additional file 20:
**Figure S1-11. Figure S1: Phenotypic and physiological responses of the two tomato genotypes under cold stress.** Figure S2: Reads distribution across genomic regions. Figure S3: The mRNA coverage analysis. Figure S4: mRNA expression profile. Figure S5: Analysis of scatterplots comparing the gene expression correlation. Figure S6: Analysis of the sample correlation according to gene expression. Figure S7: Identification of the differentially expressed genes. Figure S8: qRT-PCR validation of differentially expressed genes. Figure S9: Identification of alternative splicing. Figure S10: Small RNA reads distribution across the genomic regions. Figure S11: Clean reads mapping classification results.

## Abbreviations

A3SS: Alternative 3’ splice site
A5SS: Alternative 5’ splice site
ABA: Abscisic acid
AS: Alternative splicing events
CAT: Catalase
DEG: Differentially expressed gene
ES: Exon skipping
GO: Gene ontology
IR: Intron retention
JA: Jasmonic acid
MDA: Malondialdehyde
miRNA: microRNA
MXE: Mutually exclusive exons
PCR: Polymerase chain reaction
POD: Peroxidase
RPKM: Reads per kilobase of a gene per million reads
RLK: Receptor-like protein
SNP: Single nucleotide polymorphism
SR: Serine/arginine-rich gene
TPM: Transcripts per million
UV: Ultraviolet
