# Supplementary material for: A comparison of the low temperature transcriptomes of two tomato genotypes that differ in freezing tolerance: Solanum lycopersicum and Solanum habrochaites
Source: BMC Plant Biol. 2015 Jun 6;15:132. doi: 10.1186/s12870-015-0521-6 (PMC4458020; doi:10.1186/s12870-015-0521-6)
Supplement: Additional file 19: — Unigene sequences were assembled from S. habrochaites reads. [file 12870_2015_521_MOESM19_ESM.pdf]

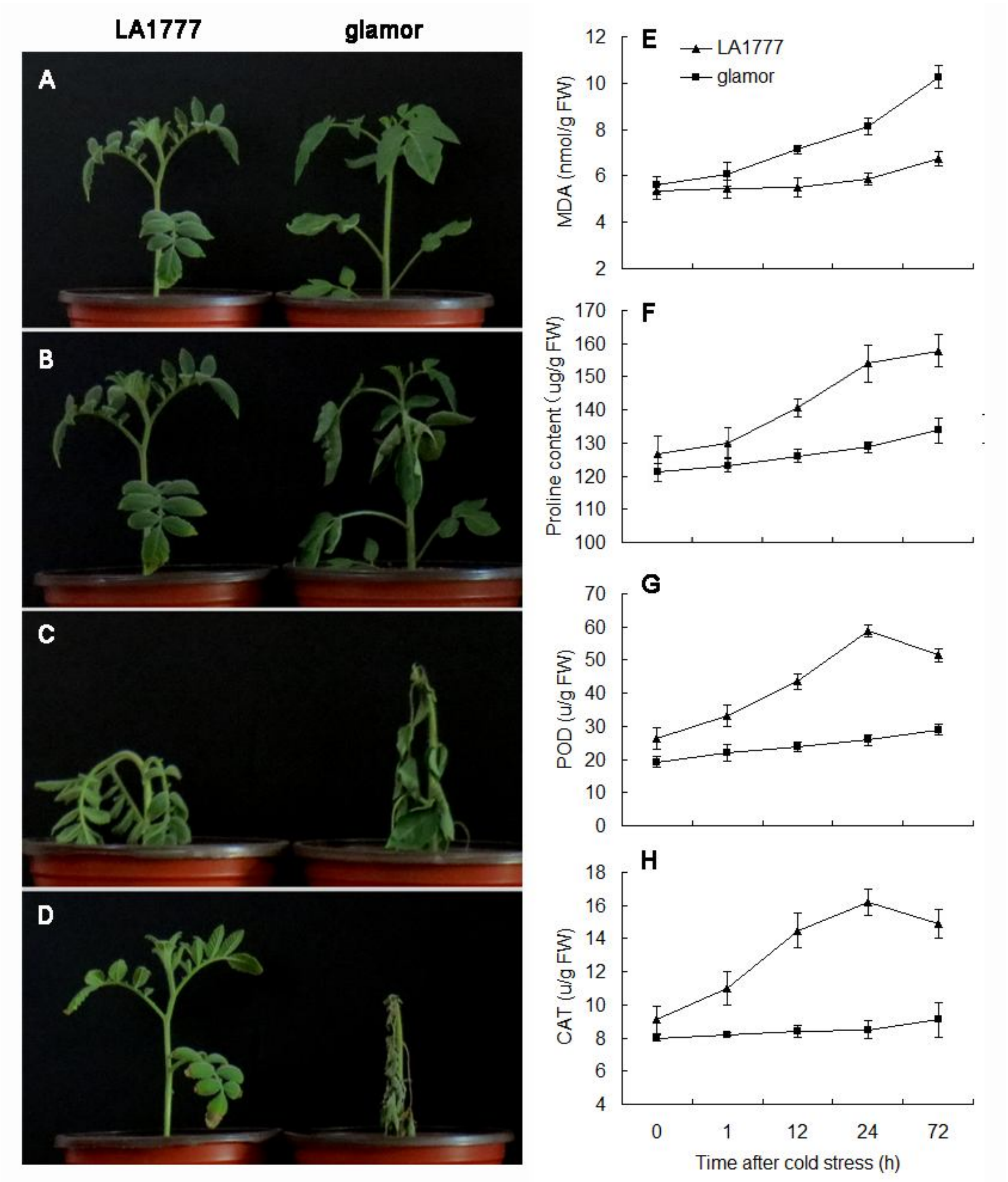

Supplemental Figure S1. Phenotypic and physiological responses of the two tomato genotypes under cold stress. Seedlings of LA1777 (*S. habrochaites*) and 'glamor' (*S. lycopersicum*) were treated as follows: control (A), treated at 4℃ for 3 d (B), treated at 4℃for 10 d (C), and recovered for 5 d after cold treatment (D). Changes in malondialdehyde (MDA) content (E), proline content (F), peroxidase (POD) activity (G), and catalase (CAT) activity (H) in the leaves of the two genotypes treated with 4℃ for 0, 1, 12, 24, and 72 h. Three independent biological replicates were used in each treatment. Data are presented as the mean ± SE of three independent biological replicates.

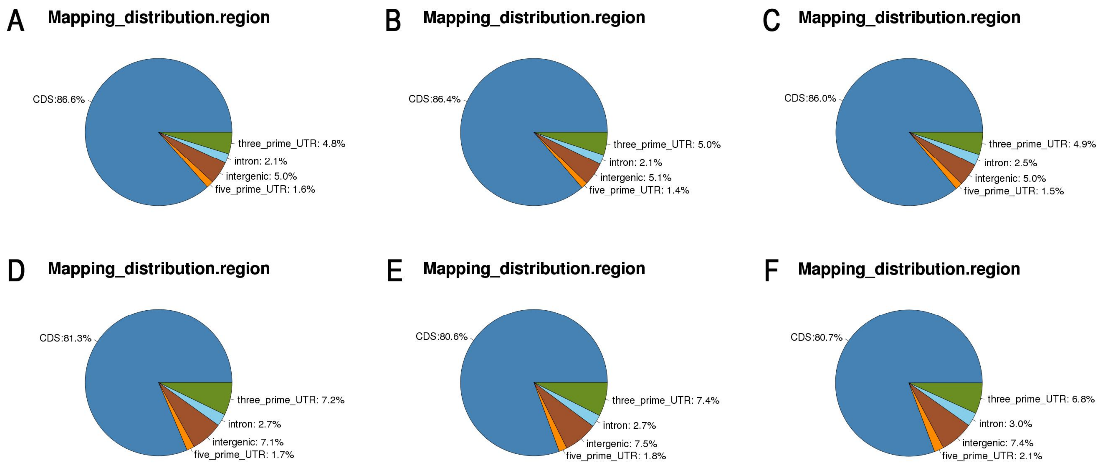

Supplemental Figure S2. Reads distribution across genomic regions of the samples Tsh0 (A), Tsh1 (B), Tsh12 (C), C0 (D), C1 (E), and C12 (F). C0, C1 and C12 represent *S. lycopersicum* cold treatment for 0h, 1h and 12h, respectively; Tsh0, Tsh1 and Tsh12 indicate *S. habrochaites* cold treatment for 0h, 1h and 12h, respectively.

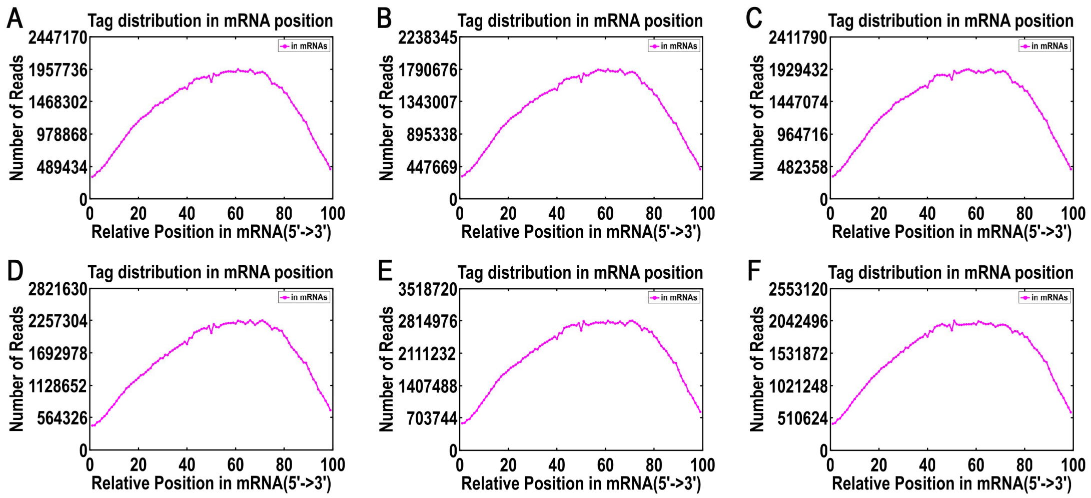

Supplemental Figure S3. The mRNA coverage analysis per hundred bins in the samples Tsh0 (A), Tsh1 (B), Tsh12 (C), C0 (D), C1 (E), and C12 (F). C0, C1 and C12 represent *S. lycopersicum* cold treatment for 0h, 1h and 12h, respectively; Tsh0, Tsh1 and Tsh12 indicate *S. habrochaites* cold treatment for 0h, 1h and 12h, respectively.

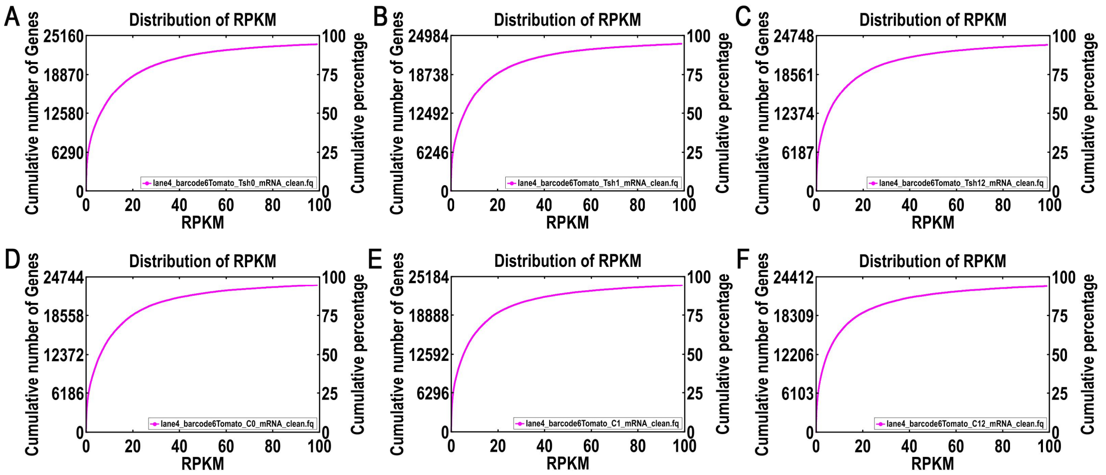

Supplemental Figure S4. mRNA expression profile of the samples Tsh0 (A), Tsh1 (B), Tsh12 (C), C0 (D), C1 (E), and C12 (F), as reflected by RPKM. C0, C1 and C12 represent *S. lycopersicum* cold treatment for 0h, 1h and 12h, respectively; Tsh0, Tsh1 and Tsh12 indicate *S. habrochaites* cold treatment for 0h, 1h and 12h, respectively.

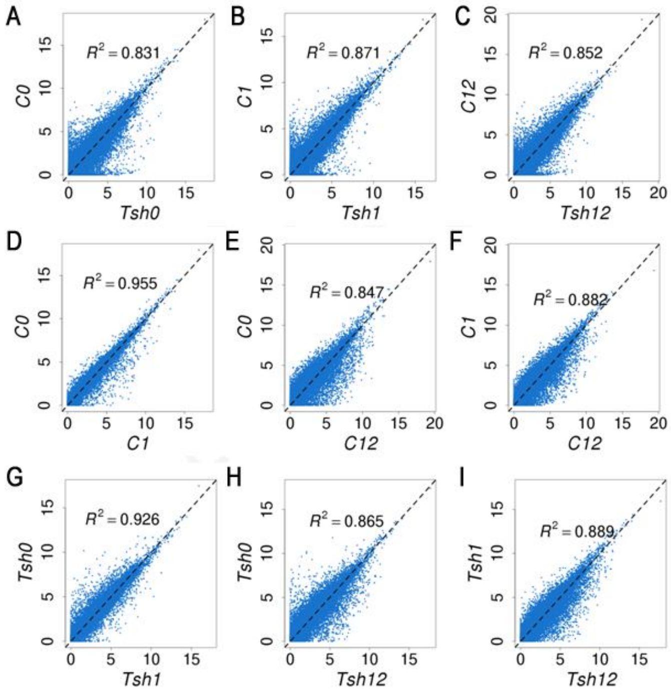

Supplemental Figure S5. Analysis of scatterplots comparing the gene expression correlation between the indicated pairs of samples Tsh0 versus C0 (A), Tsh1 versus C1 (B), Tsh12 versus C12 (C), C1 versus C0 (D), C12 versus C0 (E), C12 versus C1 (F), Tsh1 versus Tsh0 (G), Tsh12 versus Tsh0 (H), and Tsh12 versus Tsh1 (I). C0, C1 and C12 represent *S. lycopersicum* cold treatment for 0h, 1h and 12h, respectively; Tsh0, Tsh1 and Tsh12 indicate *S. habrochaites* cold treatment for 0h, 1h and 12h, respectively.

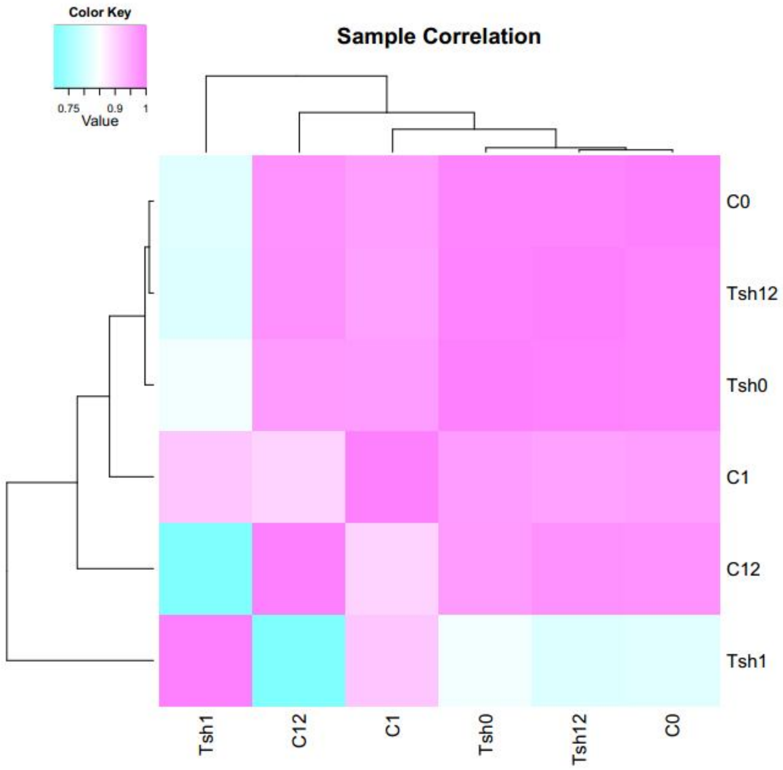

Supplemental Figure S6. Analysis of the sample correlation according to gene expression. C0, C1 and C12 represent *S. lycopersicum* cold treatment for 0h, 1h and 12h, respectively; Tsh0, Tsh1 and Tsh12 indicate *S. habrochaites* cold treatment for 0h, 1h and 12h, respectively.

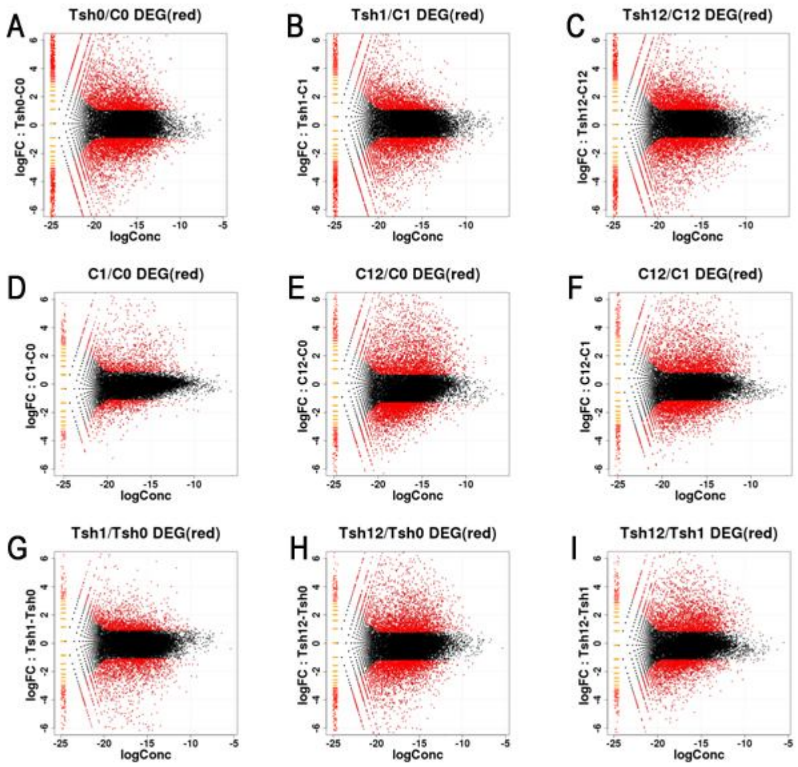

Supplemental Figure S7. Identification of the differentially expressed genes by edgeR program between the following samples: Tsh0 versus C0 (A), Tsh1 versus C1 (B), Tsh12 versus C12 (C), C1 versus C0 (D), C12 versus C0 (E), C12 versus C1 (F), Tsh1 versus Tsh0 (G), Tsh12 versus Tsh0 (H), and Tsh12 versus Tsh1 (I). DEG standard: p-value≤0.01; fold change≥2 or ≤0.5. logConc gives the overall concentration for a gene across the two groups being compared. The smear of points on the left side signifies that genes were observed in only one group of the replicate samples. C0, C1 and C12 represent *S. lycopersicum* cold treatment for 0h, 1h and 12h, respectively; Tsh0, Tsh1 and Tsh12 indicate *S. habrochaites* cold treatment for 0h, 1h and 12h, respectively.

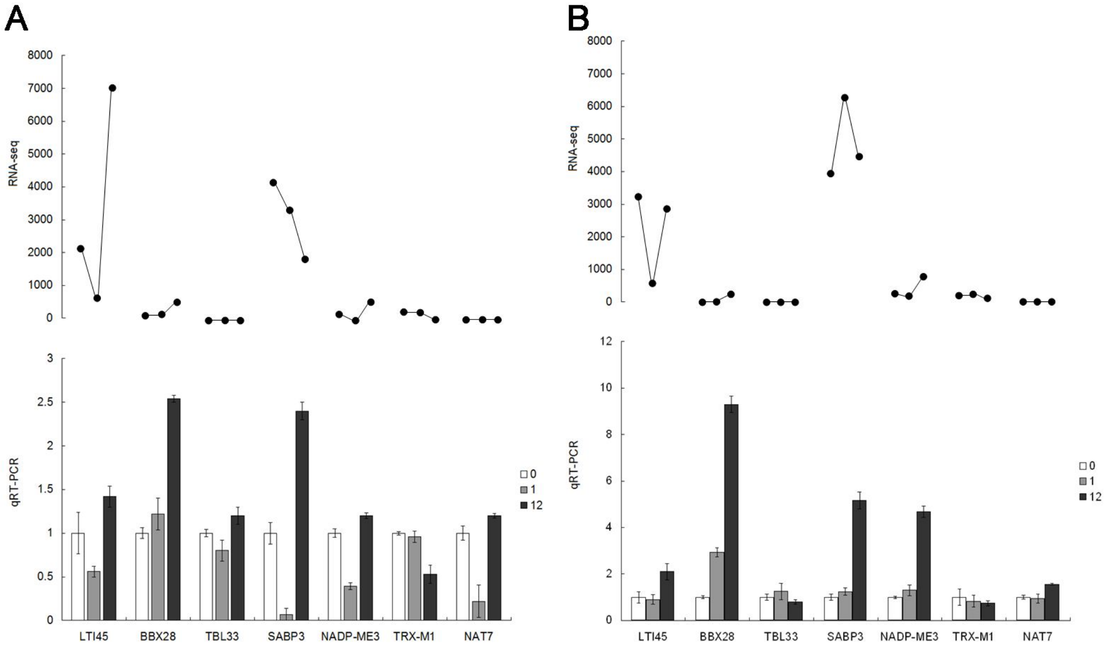

Supplemental Figure S8. qRT-PCR validation of differentially expressed genes in *S. lycopersicum* (A) and *S. habrochaites* (B) under cold. These charts showed the correlation between two types of expression profiles (RNA-seq and qRT-PCR) for the 7 ESTs. Error bars represent the SE (n = 3).

A

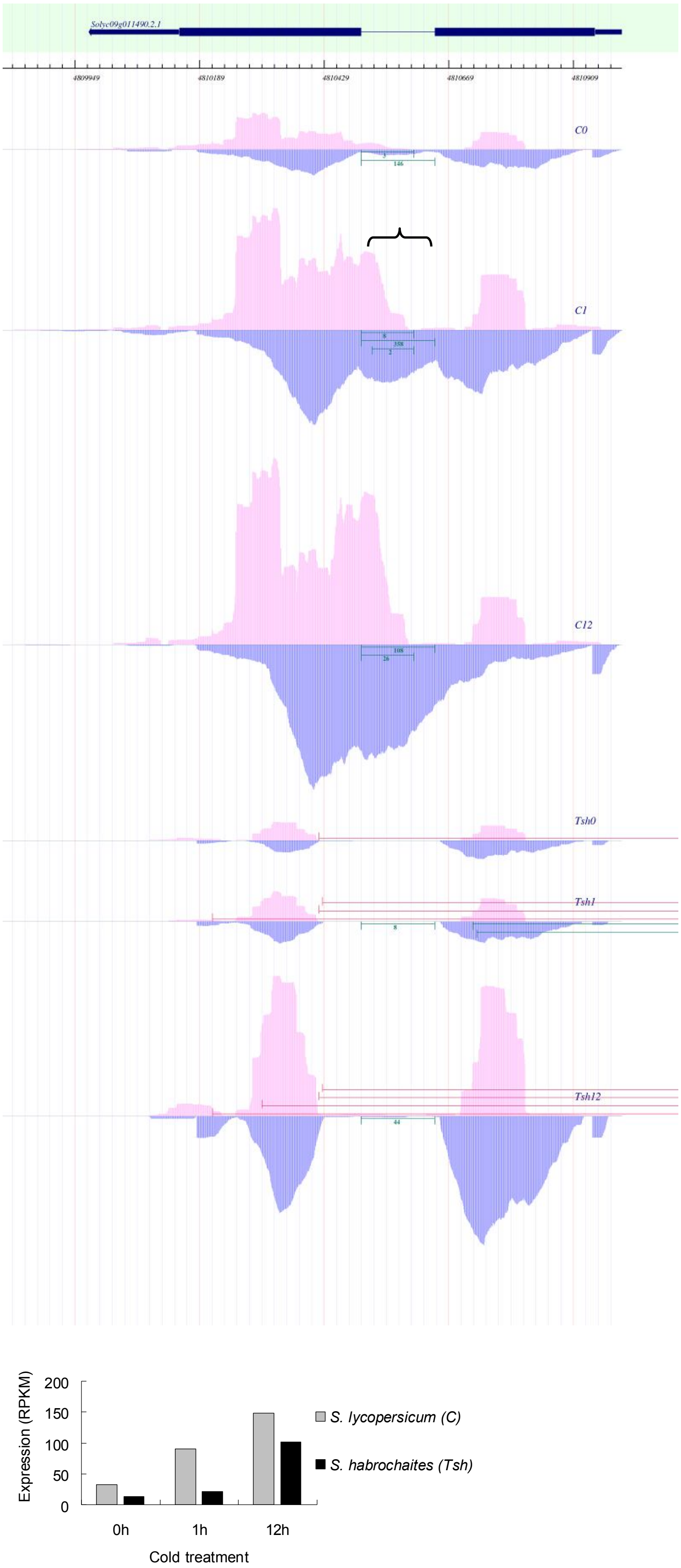

Solyc09g011490.2.1
4809949
4810189
4810429
4810669
4810909
C0
C1
C12
Tsh0
Tsh1
Tsh12
Expression (RPKM)
200
150
100
50
0
0h
1h
12h
Cold treatment
S. lycopersicum (C)
S. habrochaites (Tsh)

B

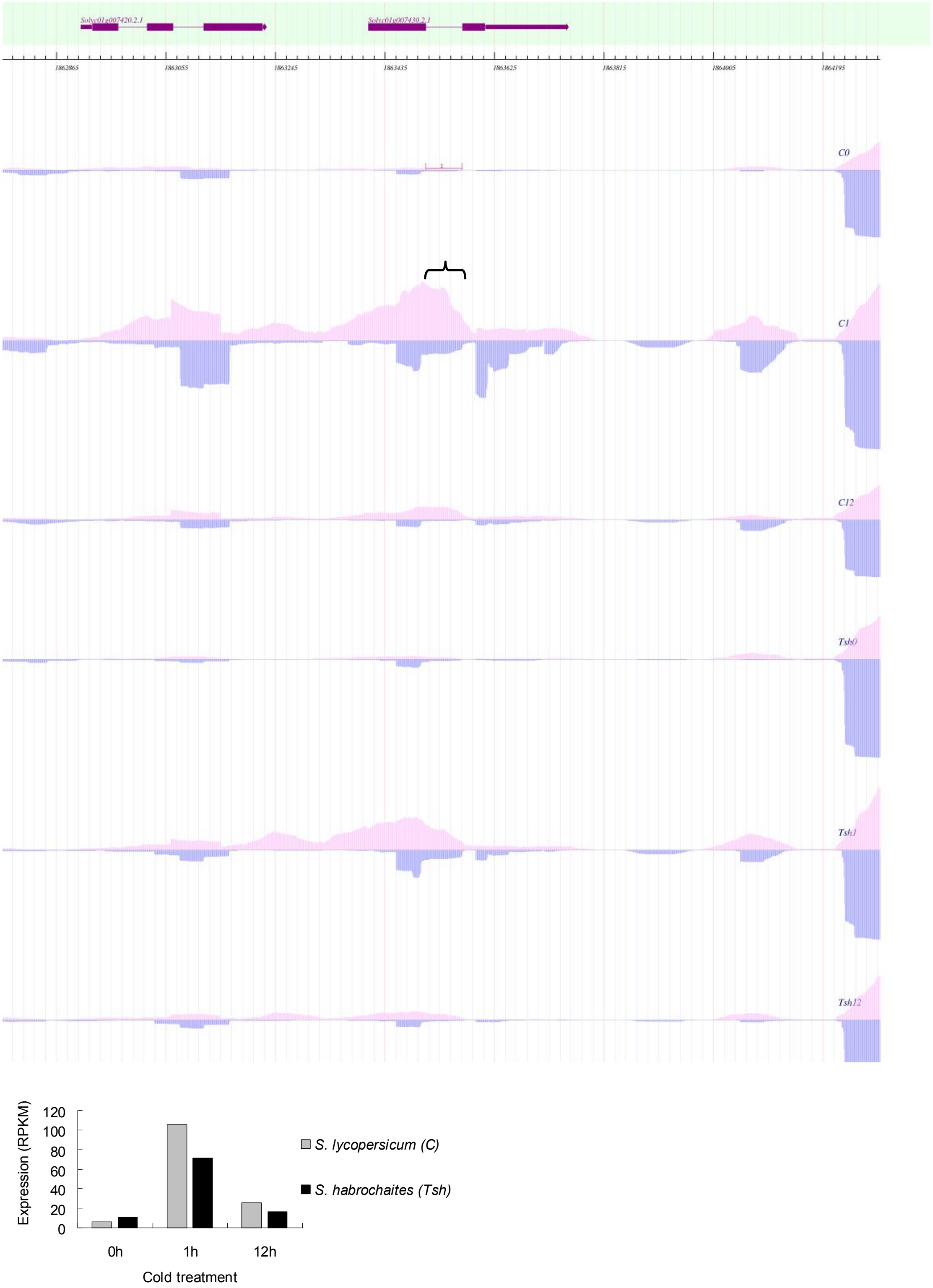

Solyc01g007420.2.1
Solyc01g007430.2.1
1862865
1863055
1863245
1863435
1863625
1863815
1864005
1864195
C0
C1
C12
Tsh0
Tsh1
Tsh12
Expression (RPKM)
120
100
80
60
40
20
0
0h
1h
12h
Cold treatment
S. lycopersicum (C)
S. habrochaites (Tsh)

C

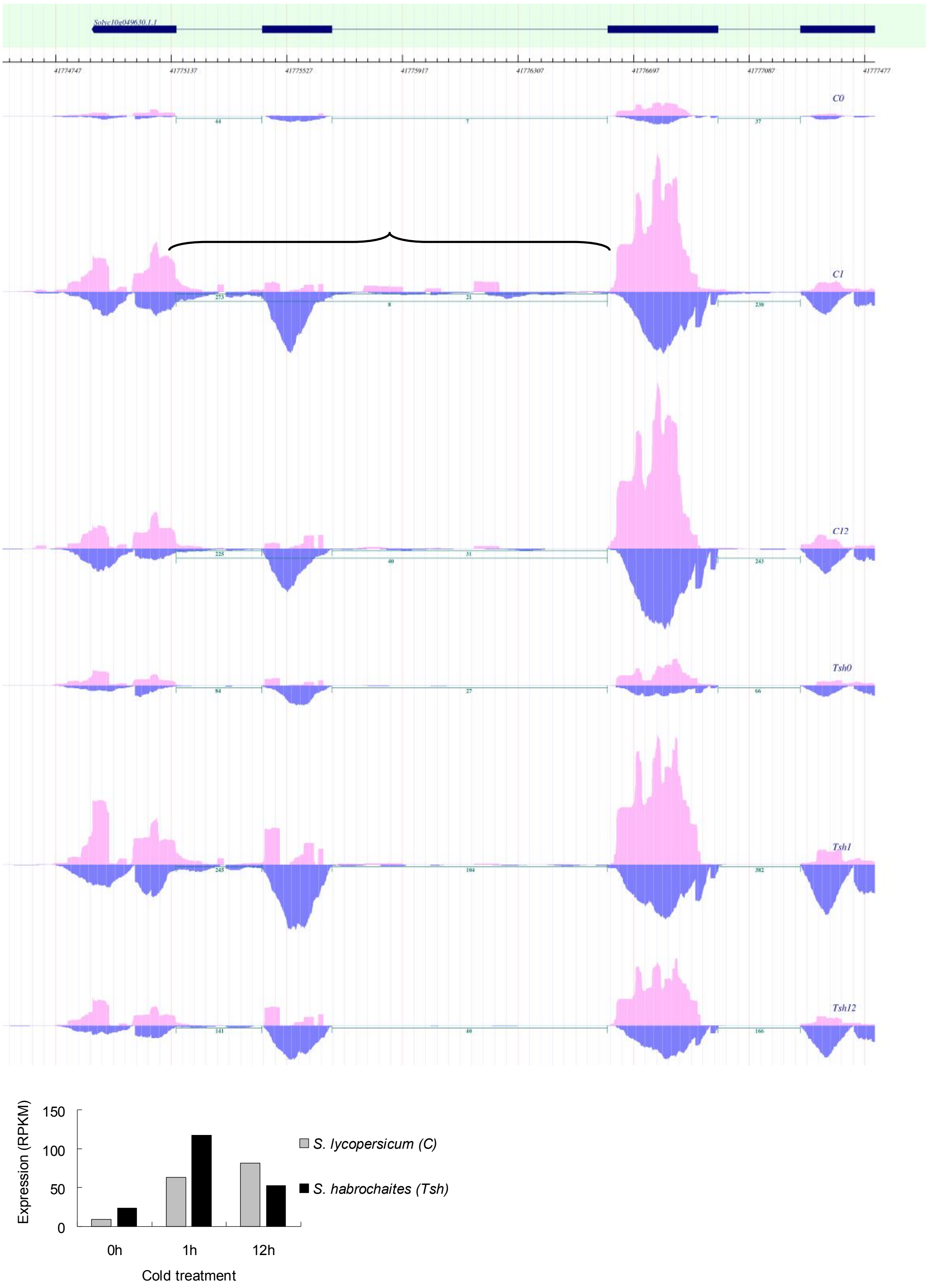

Solyc10g049630.1.1
41774747
41775137
41775527
41775917
41776307
41776697
41777087
41777477
C0
C1
C12
Tsh0
Tsh1
Tsh12
Expression (RPKM)
150
100
50
0
0h
1h
12h
Cold treatment
S. lycopersicum (C)
S. habrochaites (Tsh)

D

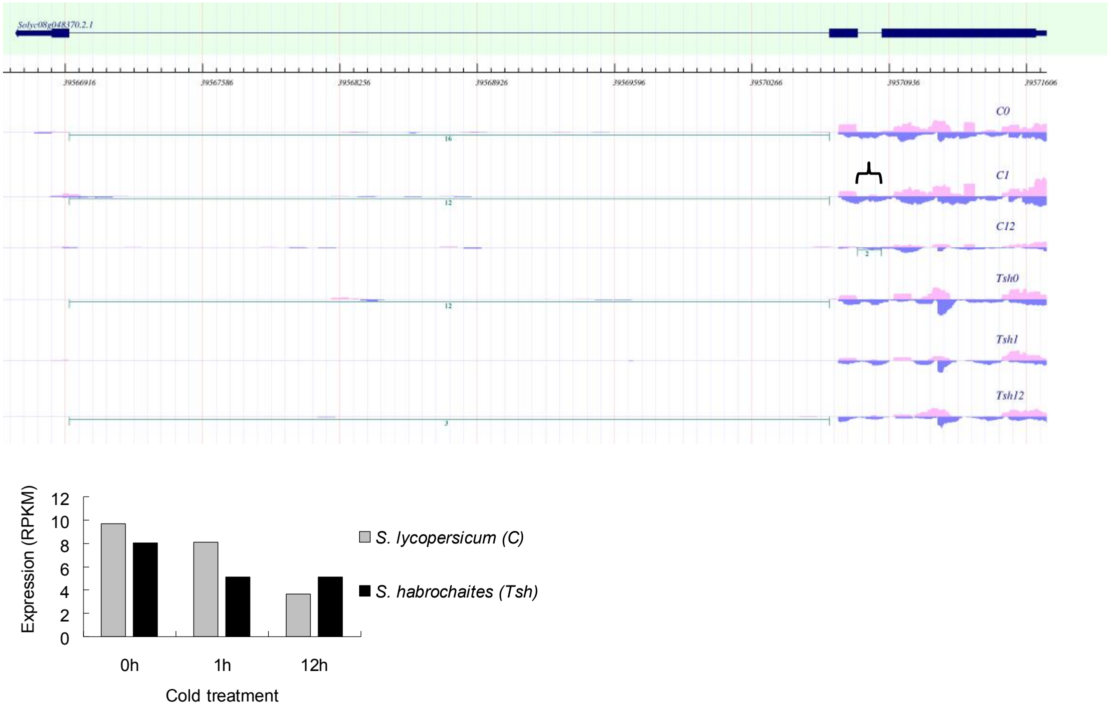
Solyc08g048370.2.1
39566916
39567586
39568256
39568926
39569596
39570266
39570936
39571606
C0
C1
C12
Tsh0
Tsh1
Tsh12
Expression (RPKM)
12
10
8
6
4
2
0
0h
1h
12h
Cold treatment
S. lycopersicum (C)
S. habrochaites (Tsh)

E

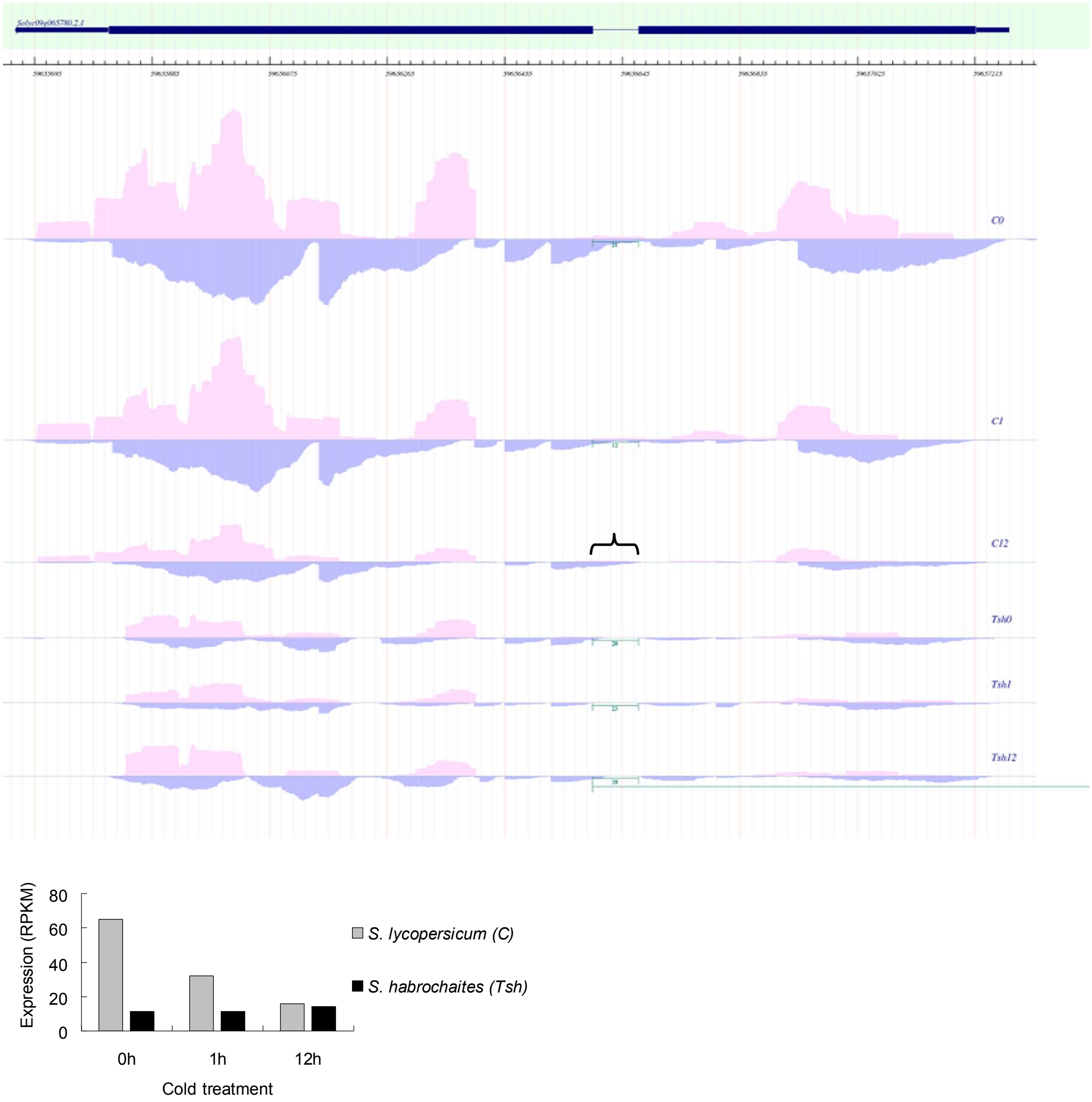
C0
C1
C12
Tsh0
Tsh1
Tsh12
Expression (RPKM)
80
60
40
20
0
0h
1h
12h
Cold treatment
S. lycopersicum (C)
S. habrochaites (Tsh)

F

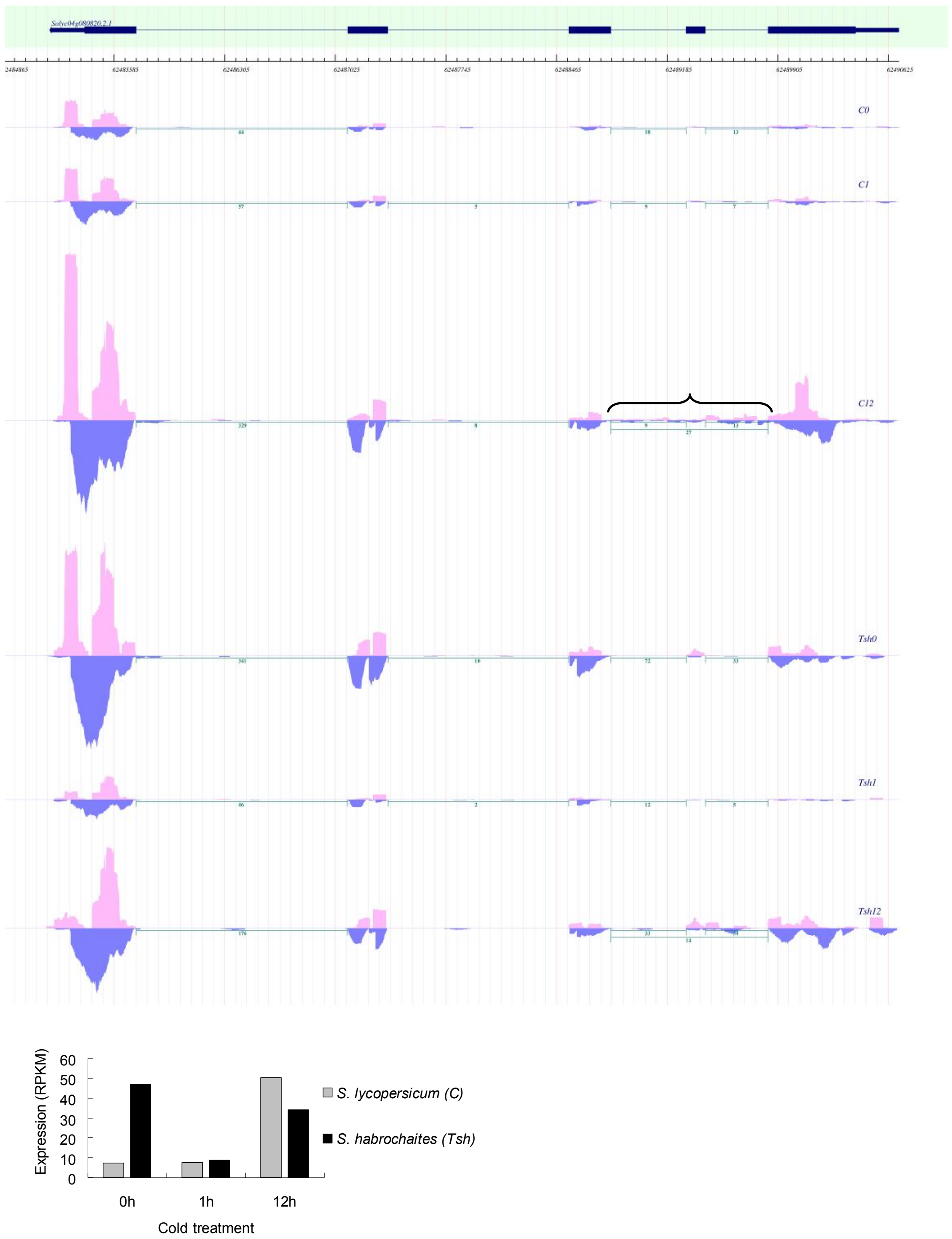

Solyc04g080820.2.1
2484865
62485585
62486305
62487025
62487745
62488465
62489185
62489905
62490625
C0
C1
C12
Tsh0
Tsh1
Tsh12
Expression (RPKM)
60
50
40
30
20
10
0
0h
1h
12h
Cold treatment
S. lycopersicum (C)
S. habrochaites (Tsh)

G

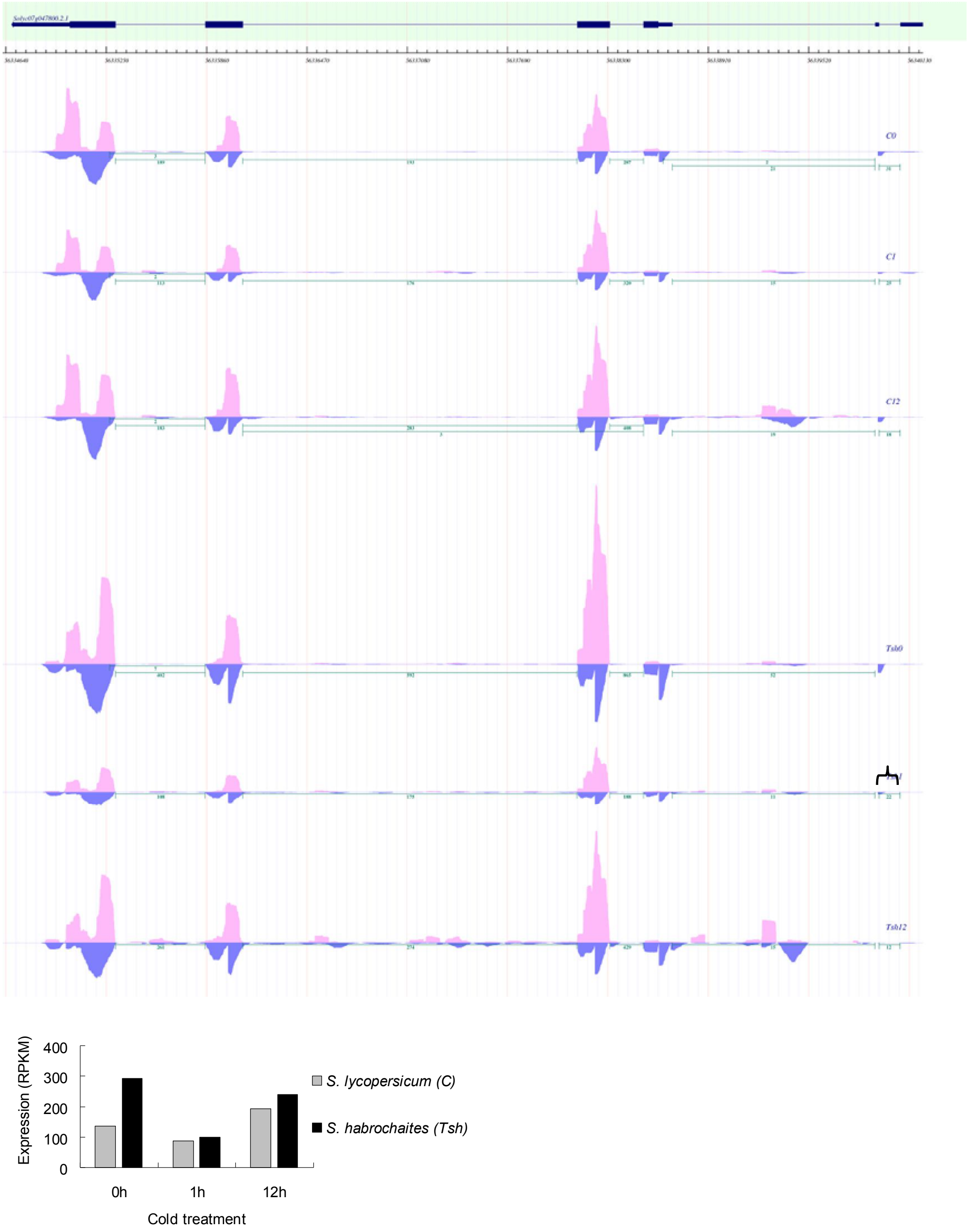
C0
C1
C12
Tsh0
Tsh12
Expression (RPKM)
400
300
200
100
0
0h
1h
12h
Cold treatment
S. lycopersicum (C)
S. habrochaites (Tsh)

H

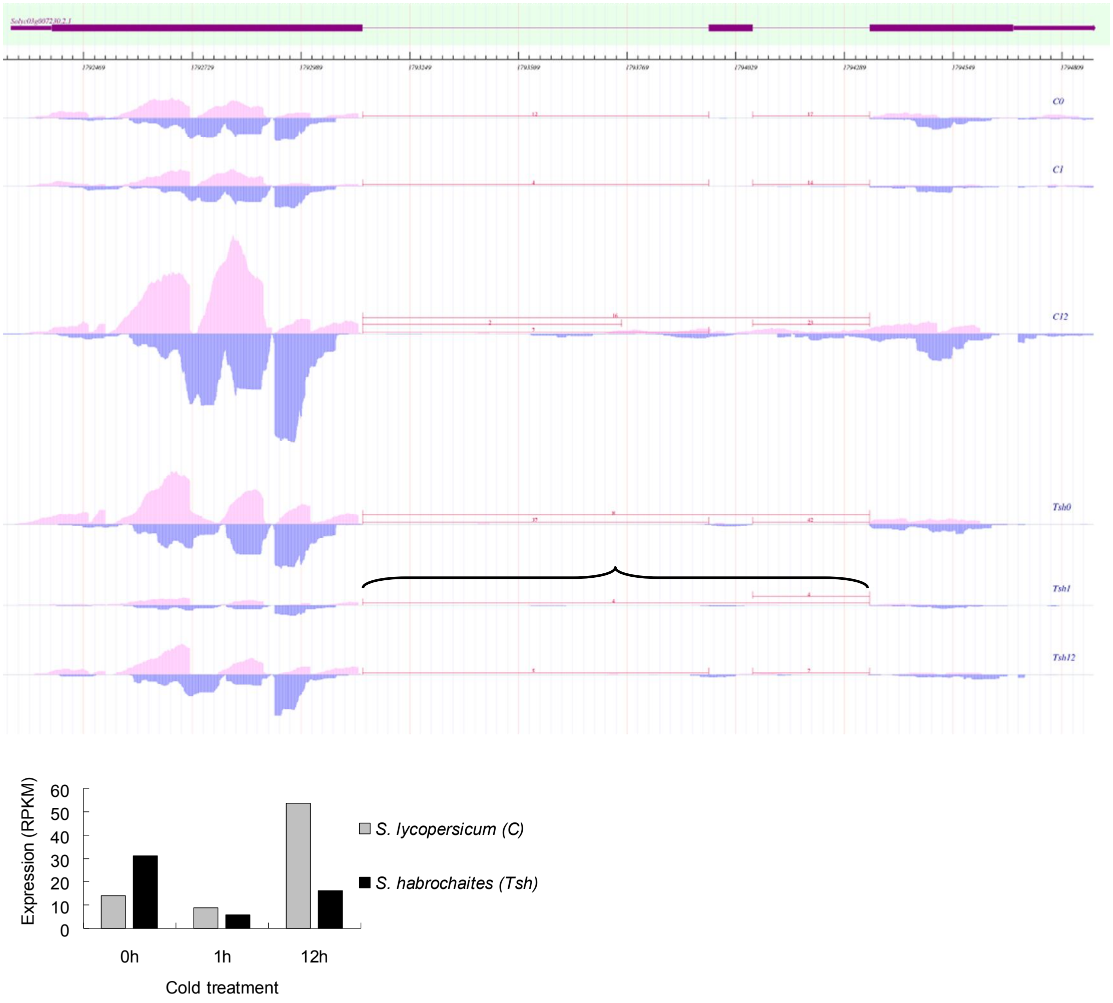
C0
C1
C12
Tsh0
Tsh1
Tsh12
Expression (RPKM)
60
50
40
30
20
10
0
0h
1h
12h
Cold treatment
S. lycopersicum (C)
S. habrochaites (Tsh)

I

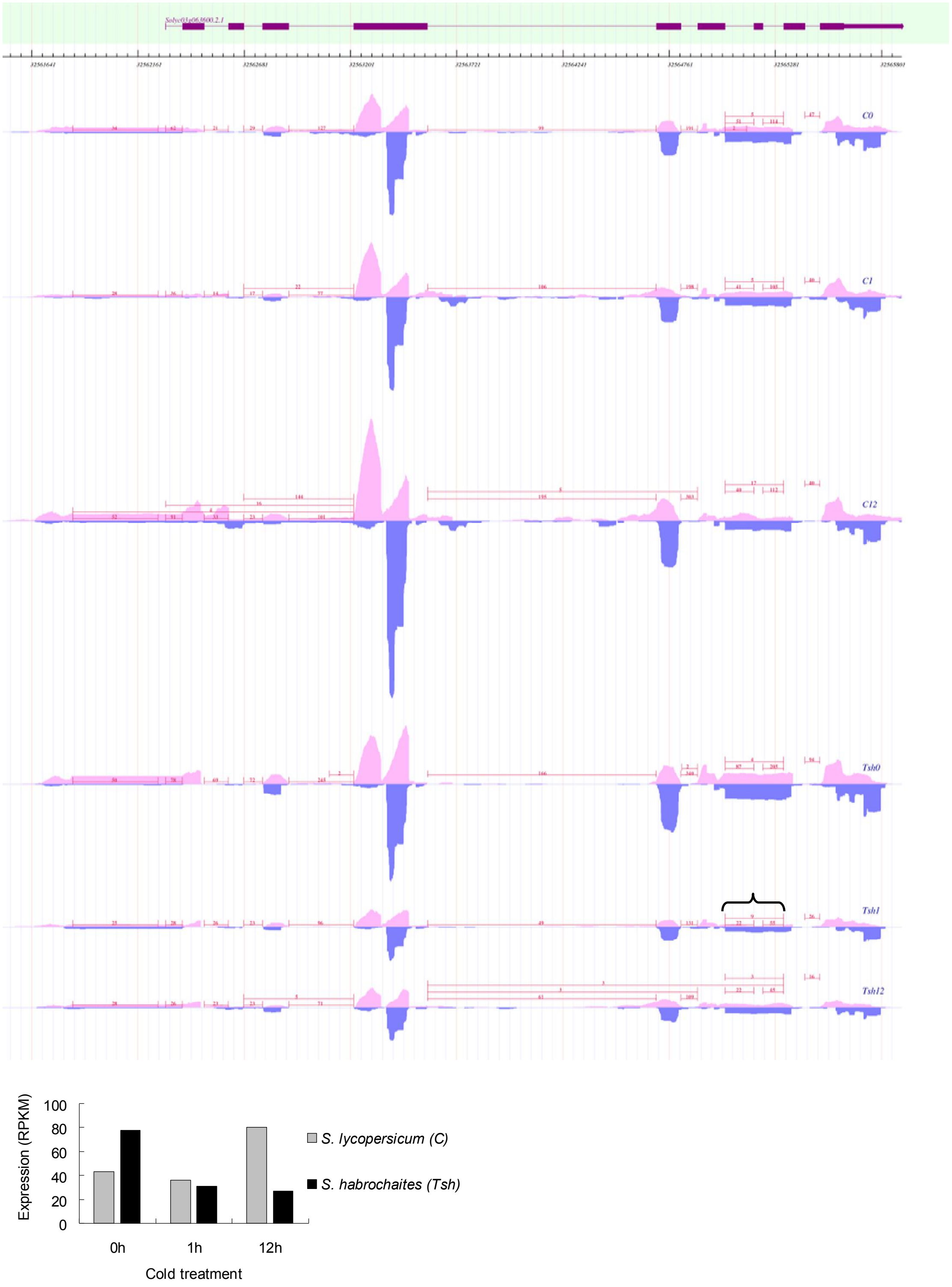
C0
C1
C12
Tsh0
Tsh1
Tsh12
Expression (RPKM)
100
80
60
40
20
0
0h
1h
12h
Cold treatment
S. lycopersicum (C)
S. habrochaites (Tsh)

J

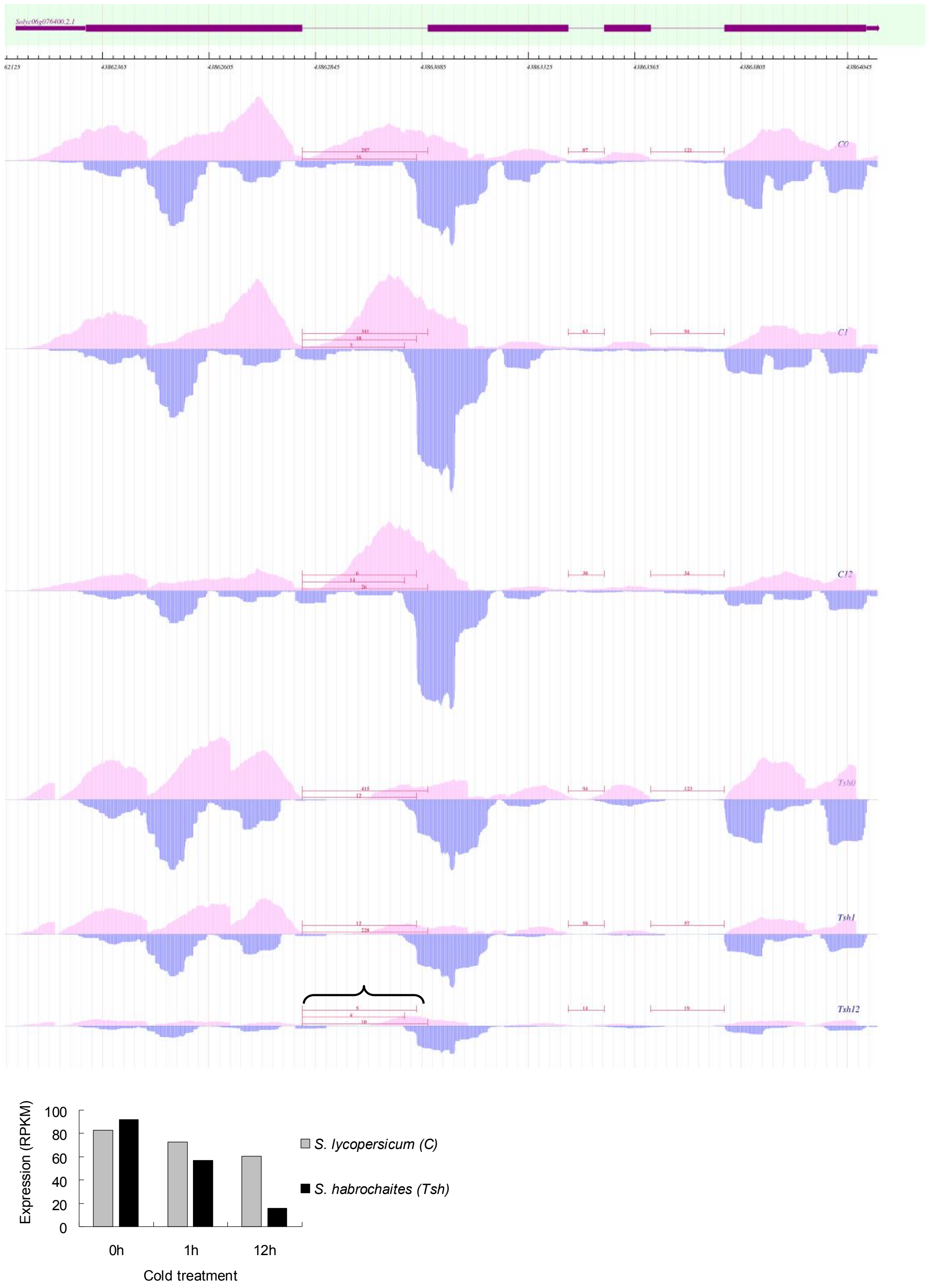

C0
C1
C12
Tsh0
Tsh1
Tsh12
Expression (RPKM)
100
80
60
40
20
0
0h
1h
12h
Cold treatment
S. lycopersicum (C)
S. habrochaites (Tsh)

K

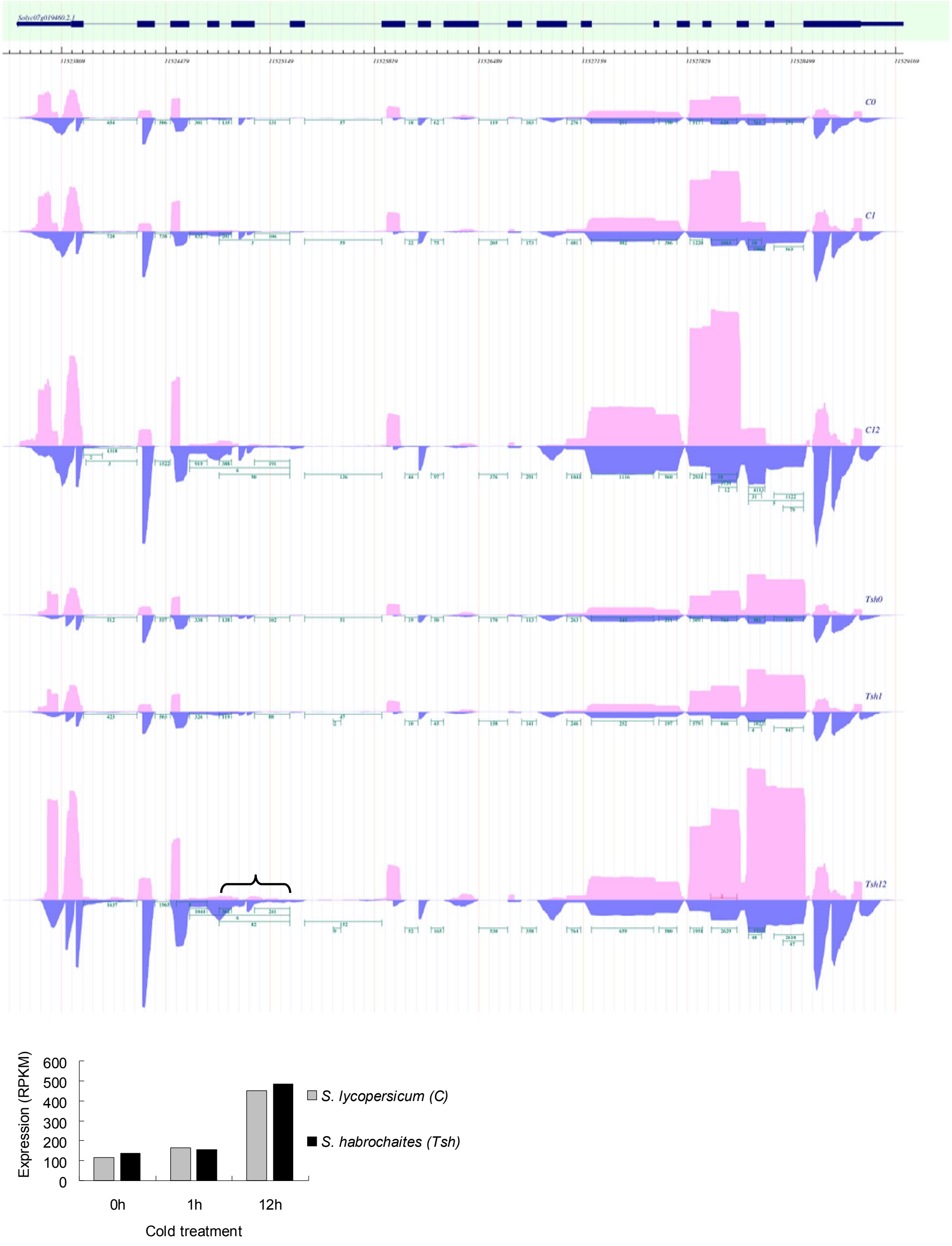
C0
C1
C12
Tsh0
Tsh1
Tsh12
Expression (RPKM)
600
500
400
300
200
100
0
0h
1h
12h
Cold treatment
S. lycopersicum (C)
S. habrochaites (Tsh)

L

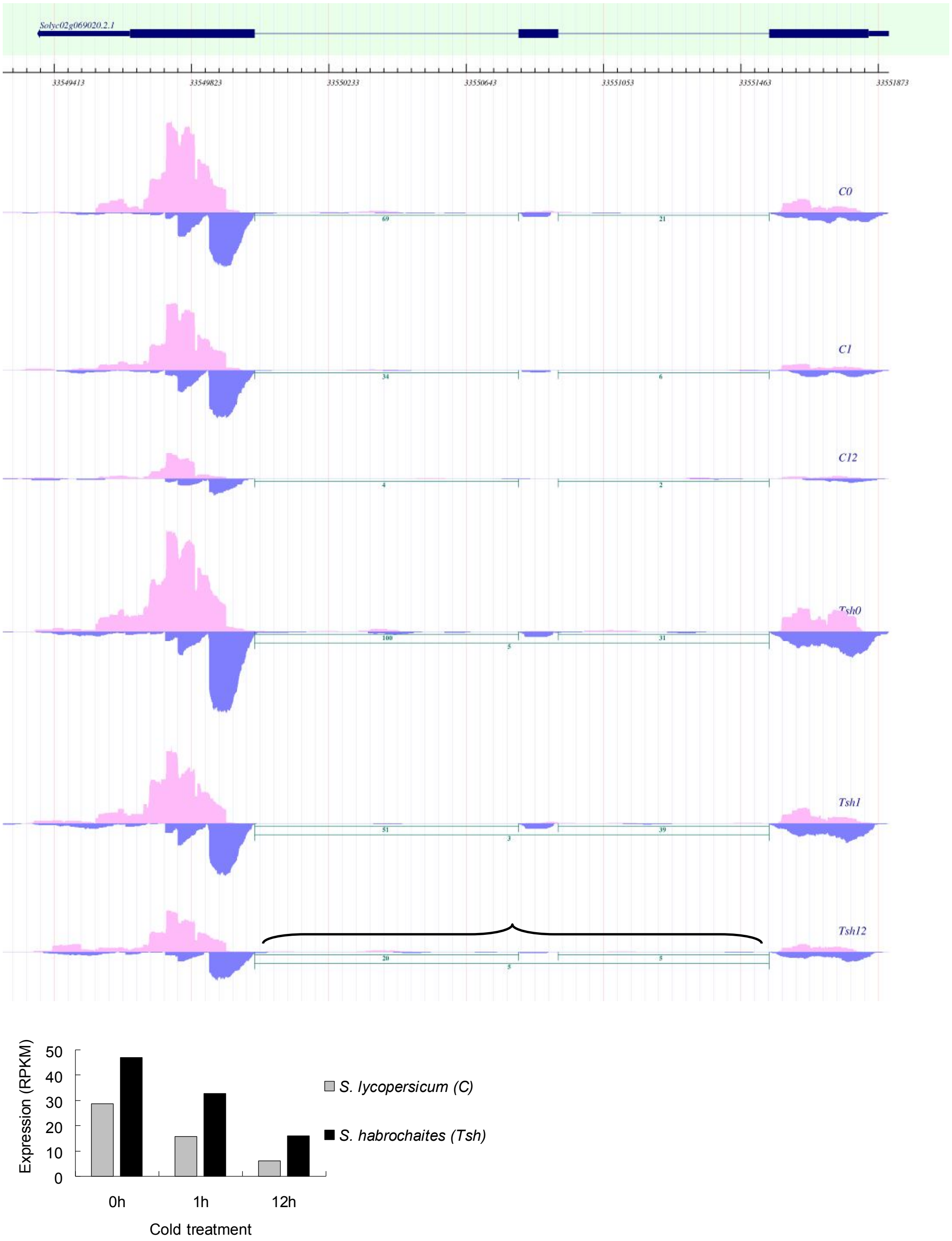
Solyc02g069020.2.1
33549413
33549823
33550233
33550643
33551053
33551463
33551873
C0
C1
C12
Tsh0
Tsh1
Tsh12
Expression (RPKM)
50
40
30
20
10
0
0h
1h
12h
Cold treatment
S. lycopersicum (C)
S. habrochaites (Tsh)

M

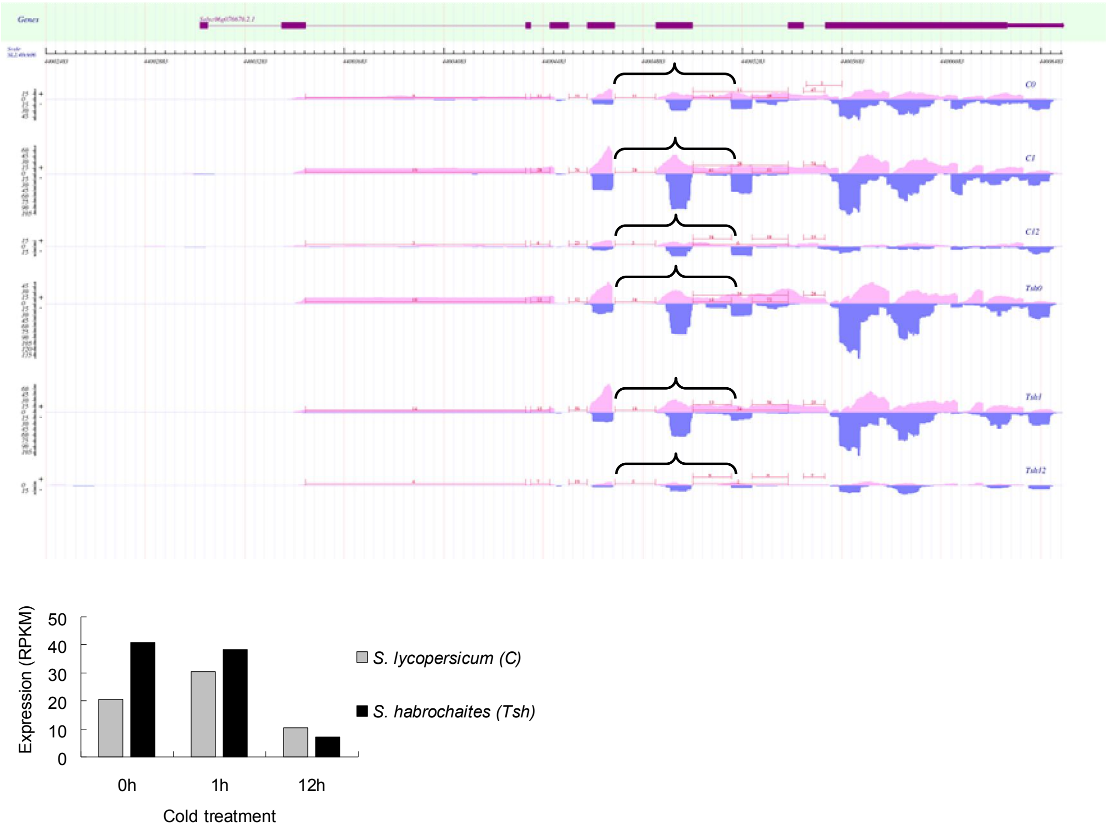

C0
C1
C12
Tsh0
Tsh1
Tsh12
Expression (RPKM)
50
40
30
20
10
0
0h
1h
12h
Cold treatment
S. lycopersicum (C)
S. habrochaites (Tsh)

N

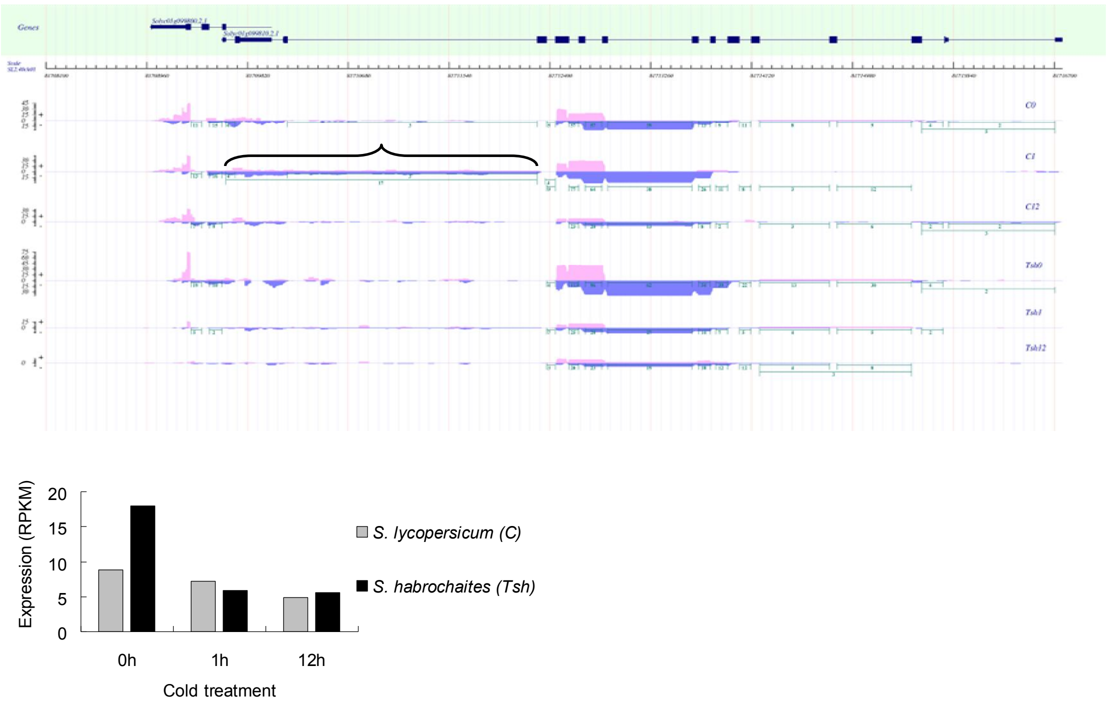

Supplemental Figure S9. Identification of alternative splicing in glutathione S-transferase TAU 8 ‘Solyc09g011490.2’ (C1 vs C0, intron retention) (A), PETG ‘Solyc01g007430.2’ (C1 vs C0, intron retention) (B), protein phosphatase 2C family ‘Solyc10g049630.1’ (C1 vs C0, exon skipping) (C), TEOSINTE BRANCHED 1, cycloidea and PCF transcription factor 2 ‘Solyc08g048370.2’ (C12 vs C0, intron retention) (D), 3-ketoacyl-CoA synthase 2 ‘Solyc09g065780.2’ (C12 vs C0, intron retention) (E), cytokinin oxidase 5 ‘Solyc04g080820.2’ (C12 vs C0, exon skipping) (F), NAD(P)-binding Rossmann-fold superfamily protein ‘Solyc07g047800.2’ (Tsh1 vs Tsh0, intron retention) (G), protein phosphatase 2CA ‘Solyc03g007230.2’ (Tsh1 vs Tsh0, exon skipping) (H), GUANYLATE KINAS 2 ‘Solyc03g063600.2’ (Tsh1 vs Tsh0, exon skipping) (I), highly ABA-induced PP2C gene 3 ‘Solyc06g076400.2’ (Tsh12 vs Tsh0, intron retention) (J), P450 reductase 2 ‘Solyc07g019460.2’ (Tsh12 vs Tsh0, exon skipping) (K), phloem protein 2-A1 ‘Solyc02g069020.2’ (Tsh12 vs Tsh0, exon skipping) (L), serine/arginine rich-like protein 45a (SR45a) ‘Solyc06g076670.2’ (C1 vs C0, C12 vs C0, Tsh0 vs Tsh1, Tsh0 vs Tsh12, cassetteExon) (M), and Serine/Arginine-Rich Protein Splicing Factor 30 (SR30) ‘Solyc01g099810.2’ (C1 vs C0, exon skipping) (N). Changes in read density coverage are indicated by pink (forward reads) and blue (reverse reads). Cold stress-associated alternative splicing events are bracketed. C0, C1 and C12 represent *S. lycopersicum* cold treatment for 0h, 1h and 12h, respectively; Tsh0, Tsh1 and Tsh12 indicate *S. habrochaites* cold treatment for 0h, 1h and 12h, respectively.

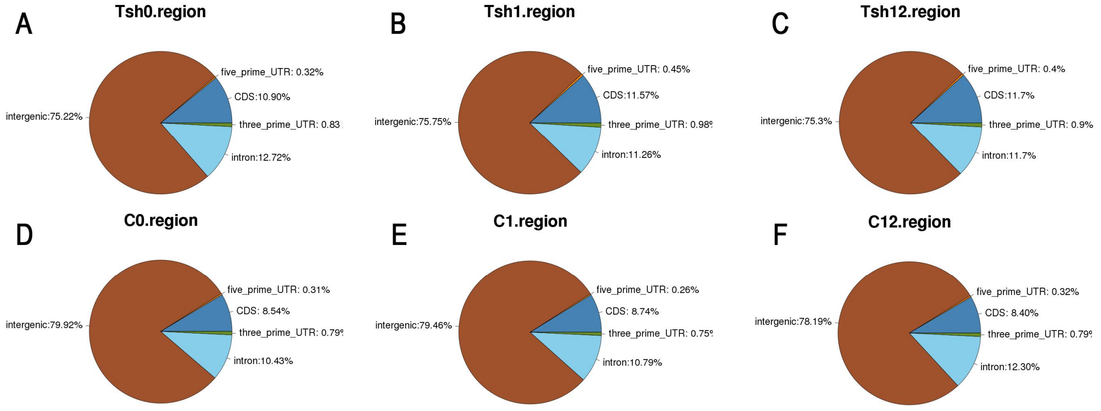

Supplemental Figure S10. Small RNA reads distribution across the genomic regions of the samples Tsh0 (A), Tsh1 (B), Tsh12 (C), C0 (D), C1 (E), and C12 (F). C0, C1 and C12 represent *S. lycopersicum* cold treatment for 0h, 1h and 12h, respectively; Tsh0, Tsh1 and Tsh12 indicate *S. habrochaites* cold treatment for 0h, 1h and 12h, respectively.

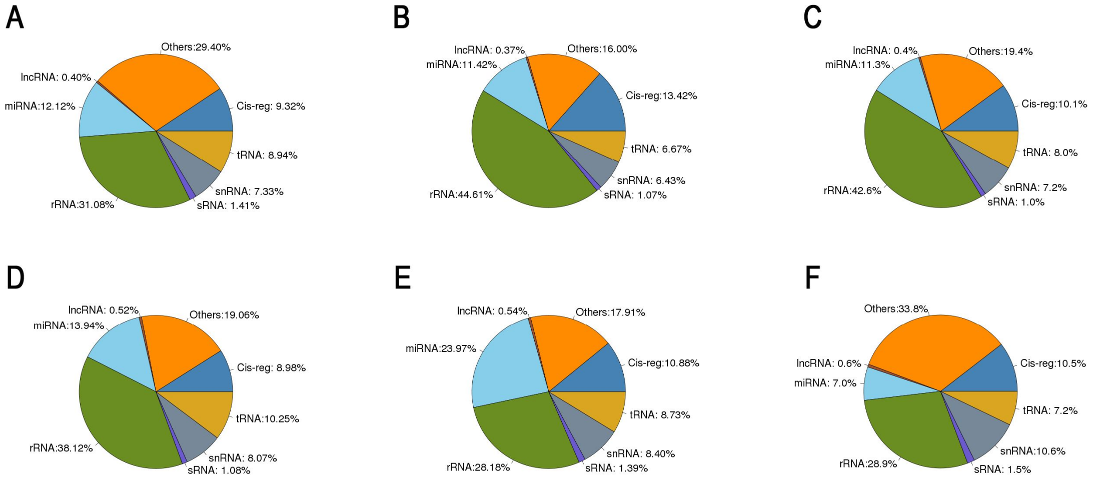

Supplemental Figure S11. Clean reads mapping classification results in Rfam of the samples Tsh0 (A), Tsh1 (B), Tsh12 (C), C0 (D), C1 (E), and C12 (F). C0, C1 and C12 represent *S. lycopersicum* cold treatment for 0h, 1h and 12h, respectively; Tsh0, Tsh1 and Tsh12 indicate *S. habrochaites* cold treatment for 0h, 1h and 12h, respectively.
